# Cellular and molecular landscapes of inflammation in anterior cruciate ligament rupture patients are independent on concurrent meniscal injury

**DOI:** 10.1186/s13075-026-03810-0

**Published:** 2026-04-18

**Authors:** Nienke J. T. van Kooten, Arjen B. Blom, Angela M. Soares, Peter M. van der Kraan, Sander Koëter, Martijn H. J. van den Bosch

**Affiliations:** 1 Experimental Rheumatology, Radboud University Medical Center, PO Box 9101, Nijmegen, 6500HB the Netherlands; 2 Orthopaedics, Canisius Wilhelmina Ziekenhuis, Nijmegen, the Netherlands

**Keywords:** Post-traumatic osteoarthritis (PTOA), Anterior cruciate ligament (ACL), Meniscus, Inflammation, Synovium, Synovial Fluid, Blood, Knee injury and osteoarthritis outcome score (KOOS)

## Abstract

**Background:**

Anterior cruciate ligament (ACL) rupture greatly increases the risk of developing knee osteoarthritis (OA), especially when accompanied by meniscal injury. Therefore, we examined biological processes in the knee joint of ACL rupture patients with or without concurrent meniscal injury. The aim of this study was to explore whether concurrent meniscal injury leads to distinct inflammatory cellular or molecular landscapes and patient reported outcome measures (PROMs), like knee function and pain, compared to an isolated ACL rupture with intact menisci.

**Methods:**

Synovium, synovial fluid and blood of 24 patients with an ACL rupture were collected at the day of ACL reconstruction surgery. Inflammation was scored in H&E-stained synovium biopsies. Cellular composition of synovium, synovial fluid and blood was determined using flow cytometry. Synovial gene expression was measured with bulk RNA sequencing. Luciferase reporter assays using SW1353 cells were used to determine signalling pathway activation in response to serum and synovial fluid. Patients completed the KOOS questionnaire preoperatively and six months and two years postoperatively, and the EQ-5D questionnaire preoperatively and two years postoperatively.

**Results:**

There was heterogeneity in synovitis among patients, but no significant differences were found between patients with an isolated ACL rupture and those with concurrent meniscal injury in terms of synovial inflammation scores, cellular composition, synovial gene expression, or pathway activation induced by serum or synovial fluid. Moreover, meniscal status did not affect PROM scores at time of ACL reconstruction. PROM scores showed generally better outcomes six months and two years postoperatively, with patients with concurrent meniscal injury showing subtly less improvement.

**Conclusion:**

The presence of concurrent meniscal injury did not influence cellular and molecular landscapes of inflammation and minimally affected patient reported outcome measures up to two years following anterior cruciate ligament reconstruction.

**Supplementary Information:**

The online version contains supplementary material available at 10.1186/s13075-026-03810-0.

## Background

Osteoarthritis (OA) is a highly prevalent joint disease with over 500 million patients worldwide. OA can affect any joint, with the knee joint being the most frequently affected as about 70% of all symptomatic OA cases is attributed to knee OA [1]. Joint trauma is a well-established risk factor for OA development, with post-traumatic osteoarthritis (PTOA) accounting for an estimated 10% of the total symptomatic knee OA cases [2].

Specifically, anterior cruciate ligament (ACL) rupture comes with a highly increased risk of knee OA development as it has been reported that up to 80% of the patients with an ACL rupture eventually show radiographic signs of OA [3, 4]. However, the mechanisms that determine why certain patients develop OA after joint trauma and others do not remain largely unclear. The injury induces a cascade of structural, mechanical and biological changes within the joint which can contribute to OA pathogenesis [5]. Notably, ACL rupture is frequently accompanied by damage to other intra-articular structures, particularly the meniscus. Previous studies have shown that the presence of meniscal injury at time of ACL reconstruction surgery comes with an increased PTOA risk, compared to ACL rupture with intact menisci [6–8]. This elevated risk can be caused by mechanical changes, as there is an altered loading on certain parts of the joint, which can affect the contact mechanics of the knee. Consequently, this altered knee joint loading may accelerate cartilage damage and joint degeneration [9, 10]. These mechanical alterations might also influence biological processes, as abnormal joint loading has been shown to stimulate the production of inflammatory mediators and catabolic enzymes by chondrocytes, also referred to as mechanoflammation, and synovial cells, further promoting cartilage breakdown [11–13]. However, whether concurrent meniscal injury at the time of ACL rupture leads to distinct inflammatory cellular and molecular landscapes, and thus possibly different treatment strategies, compared to an isolated ACL injury remains to be studied.

Following ACL rupture there is a period of acute inflammation in the knee joint that involves synovial activation and infiltration of immune cells, which together lead to the release of pro-inflammatory cytokines as well as catabolic enzymes like matrix metalloproteinases (MMPs) [14, 15]. Although the inflammatory response is most severe shortly after injury, elevated levels of some of these factors can persist in synovial fluid and tissue for extended periods of time after the initial trauma [16, 17]. Persistent low-grade inflammation may play a central role in driving the chronic joint degeneration that is characteristic of PTOA. To date, it has not been studied whether the presence of additional injuries, such as meniscus tears, impacts this inflammatory state. Understanding these potential differences would provide valuable insights into the mechanisms driving PTOA and enable more targeted prevention or treatment strategies.

In this study, we investigated whether ACL rupture with concurrent meniscal injury (AR + CMI) leads to distinct inflammatory cellular or molecular landscapes and patient reported outcome measures (PROMs), like knee function and pain, compared to an isolated ACL rupture with intact menisci (IAR).

## Methods

### Study cohort

Synovial tissue biopsies and synovial fluid of 24 ACL rupture patients were obtained during ACL reconstruction surgery at the Canisius Wilhelmina Ziekenhuis, a local hospital in Nijmegen. Patients gave written informed consent (METC 2019–5617). Demographic data of the patient cohort and information about the meniscal tear location and treatment is shown in Table 1. Patients in which severe chondropathy was detected on the preoperative MRI scan were excluded. Blood of these patients was collected on the same day just prior to surgery. Patients were asked to fill in the KOOS questionnaire shortly before surgery, 6 months after surgery and 2 years after surgery [18, 19]. Additionally, patients were asked to fill in the EQ-5D questionnaire shortly before surgery and 2 years after surgery [20]. At six months, 14 patients completed the questionnaires and at two years, 12 patients completed the questionnaires. The remaining patients were lost to follow-up. Synovial tissue was obtained from all patients. Synovial fluid was obtained from 12/24 patients and for 8 of these 12 patients, synovial fluid cells could be isolated. Blood for serum extraction was obtained from all patients, blood for flow cytometric analyses was obtained from 22/24 patients.

**Table 1:** Demographic data of the patient cohort

	All patients (*n* = 24)	IAR (*n* = 8)	AR + CMI (*n* = 16)
Age in years, mean (SD)	28.3 (7.0)	28.1 (8.4)	28.4 (6.6)
Female, *n* (%)	12 (50)	6 (75.0)	6 (37.5)
BMI, mean (SD)	25.2 (4.2)	25.8 (4.2)	24.9 (4.2)
Affected meniscus medial/lateral/both, *n* (%)			9 (56)/3 (19)/4 (25)
Partial meniscectomy, *n* (%)			5 (31.3)
Meniscal repair, *n* (%)			2 (12.5)

*IAR* isolated ACL rupture, *AR + CMI* ACL rupture + concurrent meniscal injury, *SD* standard deviation

### Synovial tissue processing

From a total of eight synovial tissue biopsies that were collected per patient, three were kept in RNAlater (Invitrogen, Thermo Fisher Scientific) for 24 h after which they were cut in half and either stored in RLT buffer (Qiagen) with 1% 2-Mercaptoethanol in MagNA Lyser Green Beads tubes (Roche) and used for bulk RNA sequencing or fixed for two hours in 4% formalin and used for histology. The remaining biopsies were collected in Roswell Park Memorial Institute (RPMI) 1640 medium (Thermo Fisher Scientific) supplemented with 200 U/mL penicillin and 200 µg/mL streptomycin (Lonza Biosciene) and were used for flow cytometry analyses.

### Histology

Synovial tissue biopsies were embedded in paraffin blocks, which were sectioned into 5 μm sections and mounted on glass slides. For each of the three biopsies, six or seven sections throughout the tissue were mounted. Hematoxylin-eosin (H&E) staining was performed and sections were individually scored for lining thickness, cellular infiltration and vascularisation using the scoring system established by Minten et al. [21]. Each parameter was scored on a 0–3 scale by a single experienced researcher (A.B.), who was blinded to whether patients had an isolated ACL rupture or concurrent meniscal injury.

### Cell isolation from synovial tissue, synovial fluid and blood

For enzymatic digestion, synovial tissue biopsies were minced with a scalpel and incubated in plain RPMI 1640 medium containing 0.1 mg/mL DNase (Merck) and 0.1 mg/mL Liberase TM (Roche) on a roller bank at 37 °C. After one hour of incubation, the suspension was brought through a 70 µM cell strainer to obtain a single cell suspension. Red blood cells were lysed using an erythrocyte lysis buffer containing 155 mM NH_4_Cl, 12 mM KHCO_3_ and 0.1 mM EDTA in H_2_O with a pH of 7.3.

Synovial fluid was centrifuged at 900 x g for 20 min at 4 °C. The supernatant was collected and centrifuged again at 15,000 rpm for 10 min, aliquoted and stored at −80 °C. The cell pellet that formed after the first centrifugation step was resuspended in PBS and brought through a 70 µM cell strainer to obtain a single cell solution. Red blood cells were lysed using the erythrocyte lysis buffer.

Erythrocytes in full blood were lysed twice for 15 min using the erythrocyte lysis buffer. After erythrocyte lysis, the remaining cells were washed with FACS buffer (PBS + 1% BSA (Sigma-Aldrich) + 2mM EDTA).

Cells from the synovial tissue, synovial fluid and blood were transferred to micronic tubes for flow cytometric analyses.

### Flow cytometry staining

Cells were incubated for 15 min in FACS buffer containing Fc-block (BD Biosciences). Cells were centrifuged and incubated for 15 min in the antibody mixtures as shown in Additional File 1 in FACS buffer with 50% Brilliant Stain Buffer (BD Biosciences). Cells were washed twice with PBS and incubated for 30 min in PBS containing ViaKrome808 fixable viability dye (Beckman Coulter). After washing twice, cells only used for extracellular staining were fixed with 1% paraformaldehyde. After 10 min of incubation, cells were washed and resuspended in FACS buffer until measurement. Cells that underwent additional intracellular staining were washed twice after incubation with the viability dye, followed by incubation for 30 min in eBioscience™ Fix/Perm solution. Permeabilised cells were centrifuged and incubated in Permeabilization Buffer from the same eBioscience™ kit with the antibodies for intracellular staining for 30 min. Cells were washed with Permeabilization Buffer and resuspended in FACS buffer until measurement. Cells were measured on a CytoFlex LX flow cytometer (Beckman Coulter). The gating strategy is shown in Additional File 2–4.

### RNA isolation from synovial tissue and bulk RNA sequencing

Synovial tissue was homogenized with ceramic beads using the MagNA Lyser Instrument (Roche). Protein digestion was performed with Proteinase K (Qiagen) and RNA was isolated using the RNeasy RNA Extraction Kit (Qiagen) according to the manufacturer’s protocol. DNA contamination was removed by using DNase I (Sigma-Aldrich). A total of 250 ng RNA per sample was used for the preparation of RNA sequencing libraries using the KAPA RNA HyperPrep kit with RiboErase (human/mouse/rat[HMR]) (Kapa Biosystems). In short, oligonucleotide hybridization and rRNA depletion, rRNA depletion cleanup, DNase digestion, DNase digestion cleanup, and RNA elution were performed according to protocol. Fragmentation and priming were performed at 94 °C for 6 min. First-strand synthesis, second-strand synthesis, and A-tailing were performed according to protocol. For adapter ligation, a 1.5-µM stock was used (NEXTflex DNA barcodes; Bioo Scientific). The first and second post-ligation cleanups were performed according to protocol. For library amplification, 11 cycles were used. Library amplification cleanup was performed using a 0.8× bead-based cleanup. The library size was determined using the high-sensitivity DNA bioanalyzer (Agilent Technologies), the library concentration was measured using the DeNovix double-stranded DNA (dsDNA) high-sensitivity assay. Sequencing was performed using an Illumina NextSeq 2000 instrument; 59-bp paired-end reads were generated.

### SW1353 cell culture and luciferase reporter cell assay

Human chondrosarcoma SW1353 reporter cell lines were generated and cryopreserved as described previously [22]. An overview of the different reporters that were used is shown in Additional File 5. SW1353 reporter cell lines were thawed and cultured in Dulbecco’s Modified Eagle Medium (DMEM)/F12 medium (Thermo Fisher Scientific) supplemented with 5% FCS (Sigma-Aldrich), 100 U/mL penicillin, 100 µg/mL streptomycin and 1 µg/mL puromycin (Merck) in a humidified atmosphere containing 5% CO_2_ at 37 °C. Cells were expanded for at least 2 passages and were used at passage 6–20. Cells were trypsinised and seeded in white flat bottom 96 well plates (Greiner) at 15,000 cells per well and serum-starved overnight in DMEM/F12 with 0% FCS, 100 U/mL penicillin and 100 µg/mL streptomycin. Serum-starved cells were stimulated in triplicate with 10% patient serum or either 10% or 1% patient synovial fluid. Reporter cell lines were functionally validated with known positive stimuli (Additional File 6). After 6 h of stimulation, supernatant was removed and cells were stored overnight at −20 °C. Cells were lysed using 30 ul ultrapure H_2_O and 30 ul Nano-Glo (Promega) was added to the cell lysate. Luminescence was measured using the CLARIOstar microplate reader (BMG Labtech).

### Data analysis

Differences between groups were analysed with GraphPad Prism software v10.4.1. A multiple unpaired t-test with the two-stage step-up method of Benjamini, Krieger and Yekutieli and a false discovery rate (FDR) of 5% was used when comparing multiple variables between two groups. An unpaired t-test was used when comparing a single variable between two groups. Spearman’s correlation coefficient was used to measure linear correlations. P values of < 0.05 were considered statistically significant. Flow cytometry data was analysed using Kaluza v2.3.0 software. Bulk RNA sequencing data analysis was performed using R Studio v4.3.1 software. Preprocessing of reads was done automatically by seq2science v1.0.0 using the RNA-seq workflow. Paired-end reads were trimmed with fastp v0.23.2 with default options. Genome assembly GRCh38.p13 was downloaded with genomepy 0.15.0. Reads were aligned with STAR v2.7.10b with default options. Afterwards, duplicate reads were marked with Picard MarkDuplicates v3.0.0. General alignment statistics were collected by samtools stats v1.16. Sample sequencing strandedness was inferred using RSeQC v5.0.1 in order to improve quantification accuracy. Deeptools v3.5.1 was used for the fingerprint, profile, correlation and dendrogram/heatmap plots, where the heatmap was made with options ‘--distanceBetweenBins 9000 --binSize 1000’. RNA-seq read duplication types were analyzed using dupRadar v1.28.0. Read counting and summarizing to gene-level was performed on filtered bam using HTSeq-count v2.0.2. The UCSC genome browser was used to visualize and inspect alignment. Transcripts per million (TPM) normalized gene counts were generated using genomepy based on longest transcript lengths. Quality control metrics were aggregated by MultiQC v1.14. Lowly expressed genes were filtered out using a threshold of > 0.5 TPM in at least 3 samples. Differential gene expression analysis was performed using the DESeq2 package. Pathway analysis was performed using ShinyGO v0.85 on genes with an unadjusted P value of < 0.01 and a log2FoldChange of > 0.6 or <−0.6 in the KEGG database with an FDR cutoff of 0.05.

### Large language models

During the preparation of this work, the author used ChatGPT in order to improve written text of this manuscript. After using this tool, N.K. reviewed and edited the content as needed and takes full responsibility for the content of the publication.

## Results

### Synovitis scores show high inter-patient variability in ACL rupture patients, independent of meniscus status

**Fig. 1:**
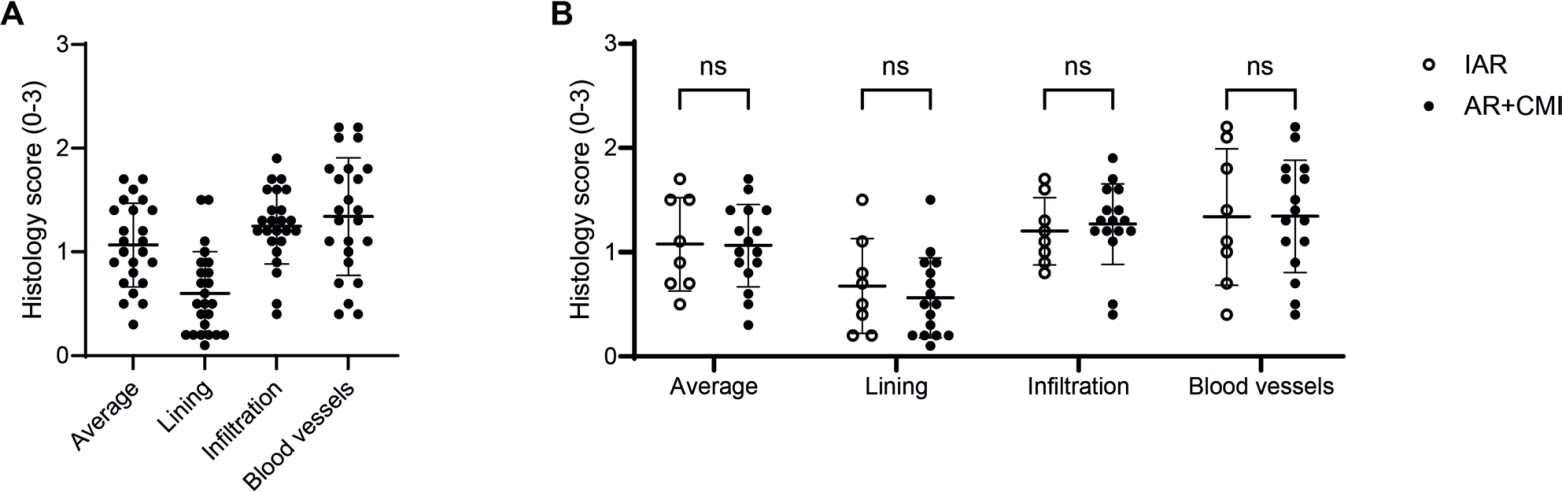
ACL rupture patients show heterogeneity in synovitis, which is not attributed to concurrent meniscal injury. **A** Synovium of ACL rupture patients was scored for various inflammatory parameters (lining thickness, infiltration and vascularisation), which showed heterogeneity in synovitis between patients. An average histology score was generated per patient by taking the mean of the three scored parameters. **B** None of the scored parameters showed a significant difference when comparing the IAR with the AR + CMI group. *n* = 24 patients (IAR: *n* = 8, AR + CMI: *n* = 16). Horizontal and vertical bars represent mean and standard deviation, respectively. IAR = isolated ACL rupture; AR + CMI = ACL rupture + concurrent meniscal injury; ns = not significant

To assess the severity and heterogeneity of synovial inflammation, we scored a variety of inflammatory features on H&E-stained tissue sections. Representative images of tissue sections with either normal (0), mild (1), moderate (2) or severe (3) scores for the various features, which are synovial lining thickness, cell infiltration and vascularisation, are shown in Additional File 7. Individual patient histology scores showed high variation between patients, as the average histology score varied from 0.3 to 1.7 (mean = 1.1; SD = 0.4), the lining score from 0.1 to 1.5 (mean = 0.6; SD = 0.4), the infiltration score from 0.4 to 1.9 (mean = 1.2; SD = 0.4) and the vascularisation score from 0.1 to 2.2 (mean = 1.3; SD = 0.6) (Fig. 1A). Next, we looked at potential differences between the IAR (*n* = 8) and AR + CMI (*n* = 16) groups. We found comparable mean scores between groups for the average (IAR = 1.1; AR + CMI=1.1), lining (IAR = 0.7; AR + CMI=0.6), infiltration (IAR = 1.2; AR + CMI=1.3) and vascularisation (IAR = 1.3; AR + CMI=1.3) scores and thus none of these parameters were significantly different between the two groups (Fig. 1B).

### Synovial tissue, synovial fluid and blood immune cell composition is not dependent on meniscus status

**Fig. 2:**
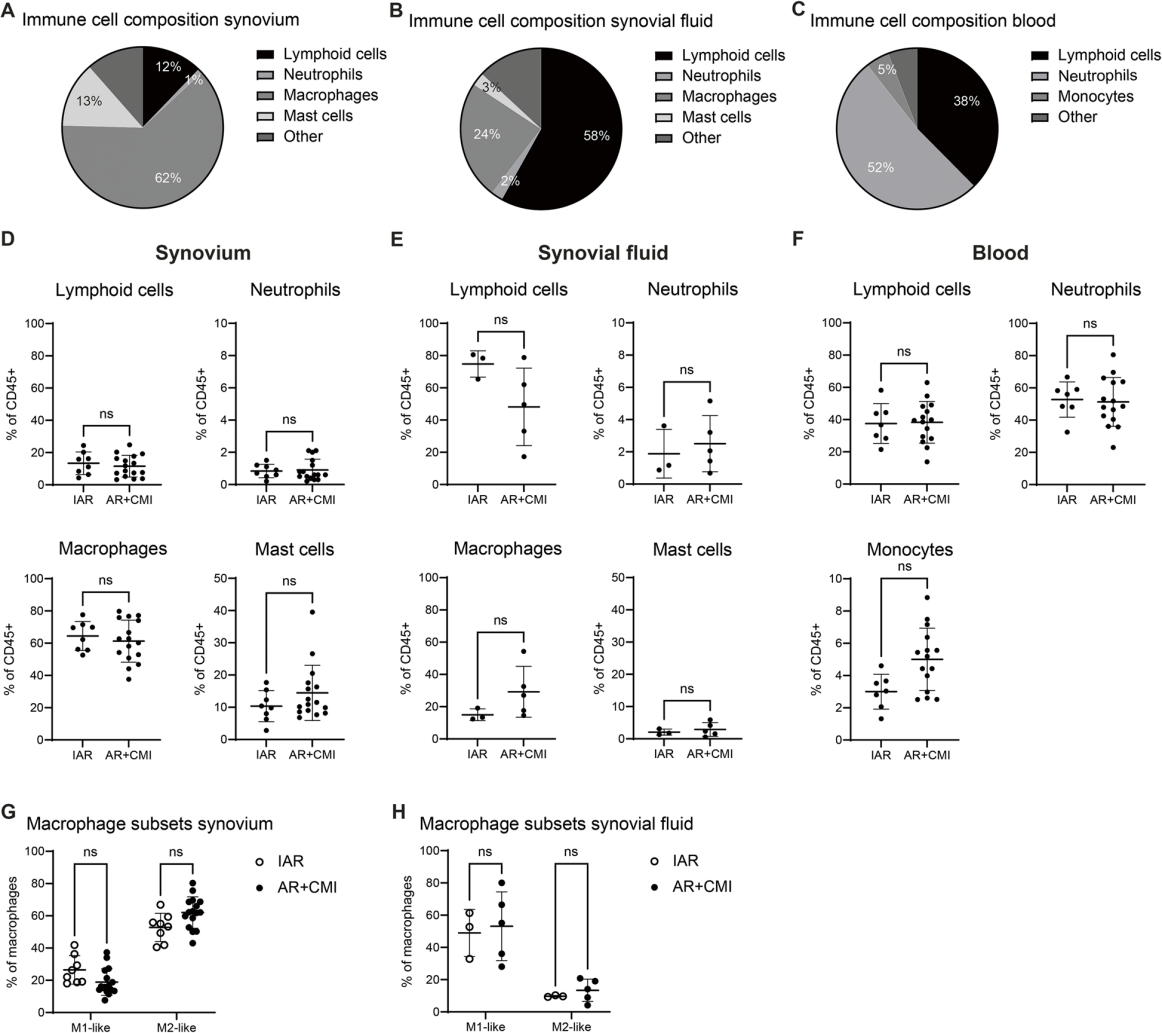
Synovial tissue, synovial fluid and blood immune cell composition are not dependent on meniscus status. **A** The immune cell composition of synovium, **B** synovial fluid and **C **blood of ACL rupture patients was determined by flow cytometry. In synovium, macrophages were the most abundant cell type, whereas in synovial fluid, the lymphoid cells were most abundant and in blood the neutrophils. **D** When comparing percentages of immune cell subtypes between the IAR and the AR + CMI group, we did not find any significant differences in cell types in the synovium, **E** synovial fluid or **F** blood. **G** When looking more specifically at macrophage subtypes, we found a higher percentage of M2-like macrophages compared to M1-like macrophages in the synovium. However, there were no differences between the IAR and AR + CMI groups regarding synovial macrophage subtypes. **H** In contrast, the synovial fluid showed a higher percentage of M1-like macrophages compared to M2-like macrophages. But also here, no differences were found between the IAR and AR + CMI groups. Synovium: *n* = 24 patients (IAR: *n* = 8, AR + CMI: *n* = 16); Synovial fluid: *n* = 8 patients (IAR: *n* = 3, AR + CMI: *n* = 5); Blood: *n* = 22 patients (IAR: *n* = 7, AR + CMI: *n* = 15). Horizontal and vertical bars represent mean and standard deviation, respectively. IAR = isolated ACL rupture; AR + CMI = ACL rupture + concurrent meniscal injury; ns = not significant

In order to get a better understanding of which immune cell types are present locally in the knee joint and systemically in the blood after an ACL tear, we next looked at the immune cell composition of the synovium, synovial fluid and blood with flow cytometry. Among the leukocytes, we found a high abundance of macrophages in the synovial tissue (62.3%), followed by mast cells (13.1%) and lymphoid cells (12.2%). Additionally, we found a very low abundance of neutrophils in the synovium (0.9%) (Fig. 2A). Interestingly, the synovial fluid immune cell composition showed a distinct pattern with lymphoid cells being most abundant (58.1%), followed by macrophages (23.9%) and mast cells (2.6%). Similar to the synovium, neutrophils were the least abundant cell type measured in the synovial fluid (2.3%) (Fig. 2B). Furthermore, the cell composition of the blood showed neutrophils as the most abundant measured cells (51.9%), followed by lymphoid cells (37.6%) and monocytes (4.7%) (Fig. 2C). Although we did observe heterogeneity between individual patients here as well, when comparing IAR with AR + CMI we did not find any differences in immune cell population percentages in either the synovium (IAR: *n* = 8; AR + CMI: *n* = 16), synovial fluid (IAR: *n* = 3; AR + CMI: *n* = 5), or blood (IAR: *n* = 7; AR + CMI: *n* = 15) (Fig. 2D-F). As macrophages were the most abundant immune cell type in the synovium and are known to be important in OA pathogenesis, we looked further into macrophage phenotype. In the synovium, we found a higher presence of anti-inflammatory M2-like macrophages (58.9% of total macrophages) compared to the presence of pro-inflammatory M1-like macrophages (21.4% of total macrophages). In the synovial fluid, on the contrary, we found a higher percentage M1-like macrophages (51.6% of total macrophages) compared to the percentage of M2-like macrophages (12.0% of total macrophages). Macrophages that were not classified as M1-like or M2-like were classified as intermediate and are not shown here. We did not observe any differences regarding macrophage subtypes between the IAR and AR + CMI groups (Fig. 2G-H).

### Synovial gene expression and pathway activation induced by serum or synovial fluid is not dependent on concurrent meniscus injury

**Fig. 3:**
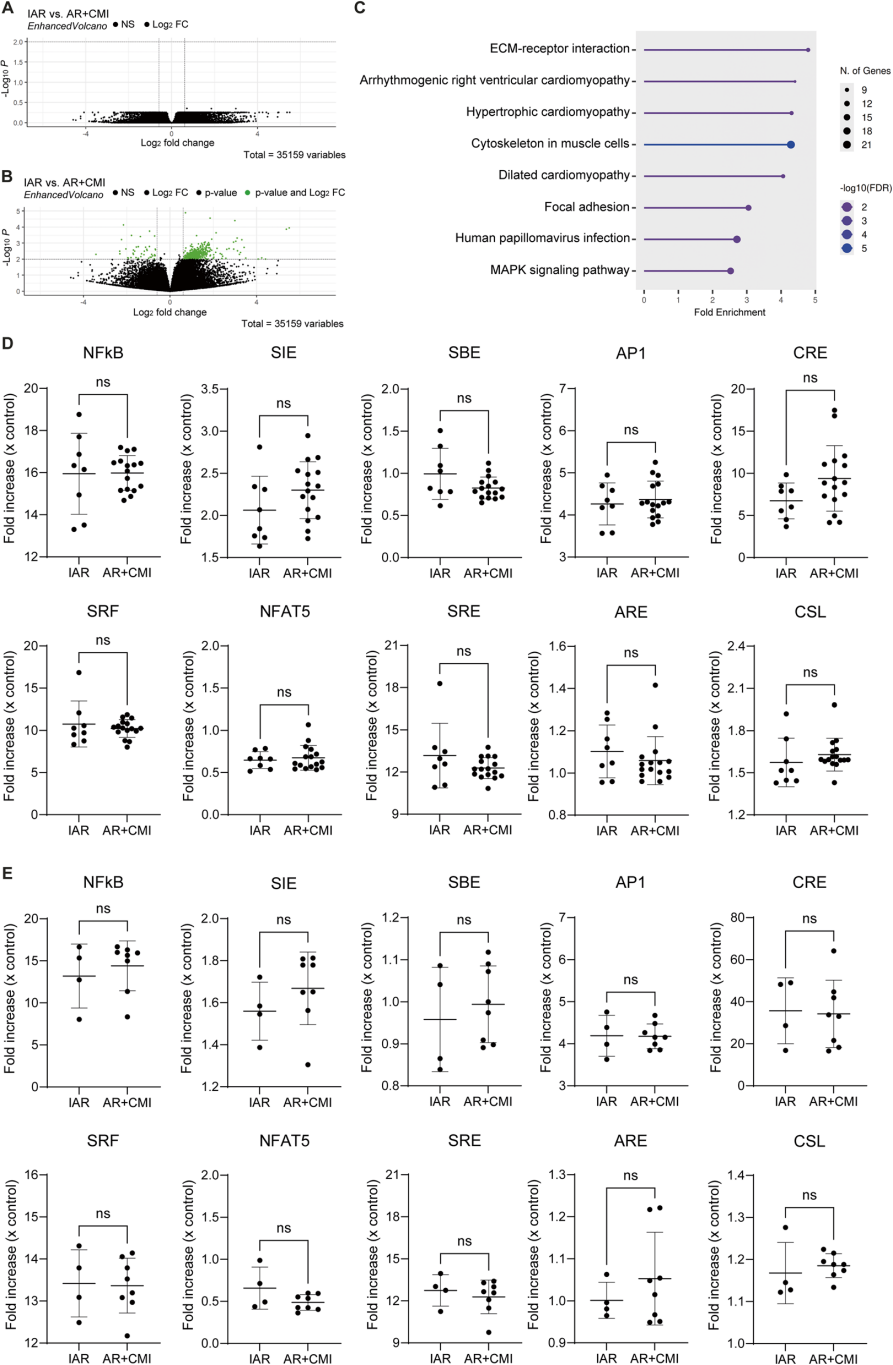
Synovial gene expression and pathway activation induction is independent on concurrent meniscal injury. **A** Volcano plot of the differential gene expression analysis of IAR vs. AR + CMI patient groups based on the adjusted p value. The adjusted p value did not show any significant differentially expressed genes between these groups. **B** Volcano plot of the differential gene expression analysis of IAR vs. AR + CMI patient groups based on the unadjusted p value. Genes with an unadjusted p value of < 0.01 and a log2FoldChange of > 0.6 or <−0.6 were selected for the pathway analysis and shown in green. **C** Pathway analysis resulted in eight pathways with an FDR of < 0.05 that contained between 9–21 of the selected genes. **D** Intracellular pathway activation induced by serum or **E**) synovial fluid measured by a luciferase reporter cell-based assay. For all ten reporter cell lines, there were no differences in pathway activation between patients with an isolated ACL rupture and patients with concurrent meniscal injury for both serum and synovial fluid. Bulk RNA sequencing and serum-induced reporter assays: *n* = 24 patients (IAR: *n* = 8, AR + CMI: *n* = 16); Synovial fluid-induced reporter assays: *n* = 12 patients (IAR: *n* = 4, AR + CMI: *n* = 8). Dotted vertical bars represent the log2FoldChange cutoff values of 0.6 and − 0.6. Dotted horizontal bars represent the p value cutoff of 0.01. Solid horizontal and vertical bars represent mean and standard deviation, respectively. IAR = isolated ACL rupture; AR + CMI = ACL rupture + concurrent meniscal injury; ns = not significant. A detailed overview of the reporter cell lines is shown in Additional File 5

As we did not observe any differences in synovium histology inflammation scores and immune cell composition of synovium, synovial fluid and blood between the IAR and AR + CMI groups, we next evaluated differences in synovial activation state by evaluating synovial gene expression. Bulk RNA sequencing of the synovium did not show any significantly differentially regulated genes between patients with an ACL rupture and concurrent meniscal injury (*n* = 16) and patients with an isolated ACL rupture (*n* = 8) as none of the adjusted p-values reached the threshold of < 0.05 (Fig. 3A). As we observed no differences in individual gene expression, we next performed a pathway analysis on the genes with an unadjusted p-value of < 0.01 and a log2FoldChange of > 0.6 or <−0.6 to look at possible pathway activation. Figure 3B shows a volcano plot of the gene selection for the pathway analysis. We identified eight pathways with an FDR of < 0.05 which contained between 9 and 21 of the selected genes (Fig. 3C). An overview of these pathways with the specific genes highlighted is shown in Additional File 8. To study intracellular pathway activation more precisely, we used a luciferase reporter cell-based assay to measure whether the mix of (inflammatory) factors present in serum (IAR: *n* = 8; AR + CMI: *n* = 16) or synovial fluid (IAR: *n* = 4; AR + CMI: *n* = 8) of the patients can induce activation of specific intracellular pathways. We observed an activation of several pathways (NFκB, SIE, AP1, CRE, SRF, SRE and CSL) by both serum and synovial fluid, while the other pathways (SBE, NFAT5 and ARE) showed no or very little activation with an average fold increase of < 1.1. Although various pathways showed activation, we did not observe differences in pathway activation between patients with an isolated ACL rupture and patients with concurrent meniscus injury (Fig. 3D-E).

### Patients with concurrent meniscal injury do not show worse patient reported outcome measures at time of ACL reconstruction

**Fig. 4:**
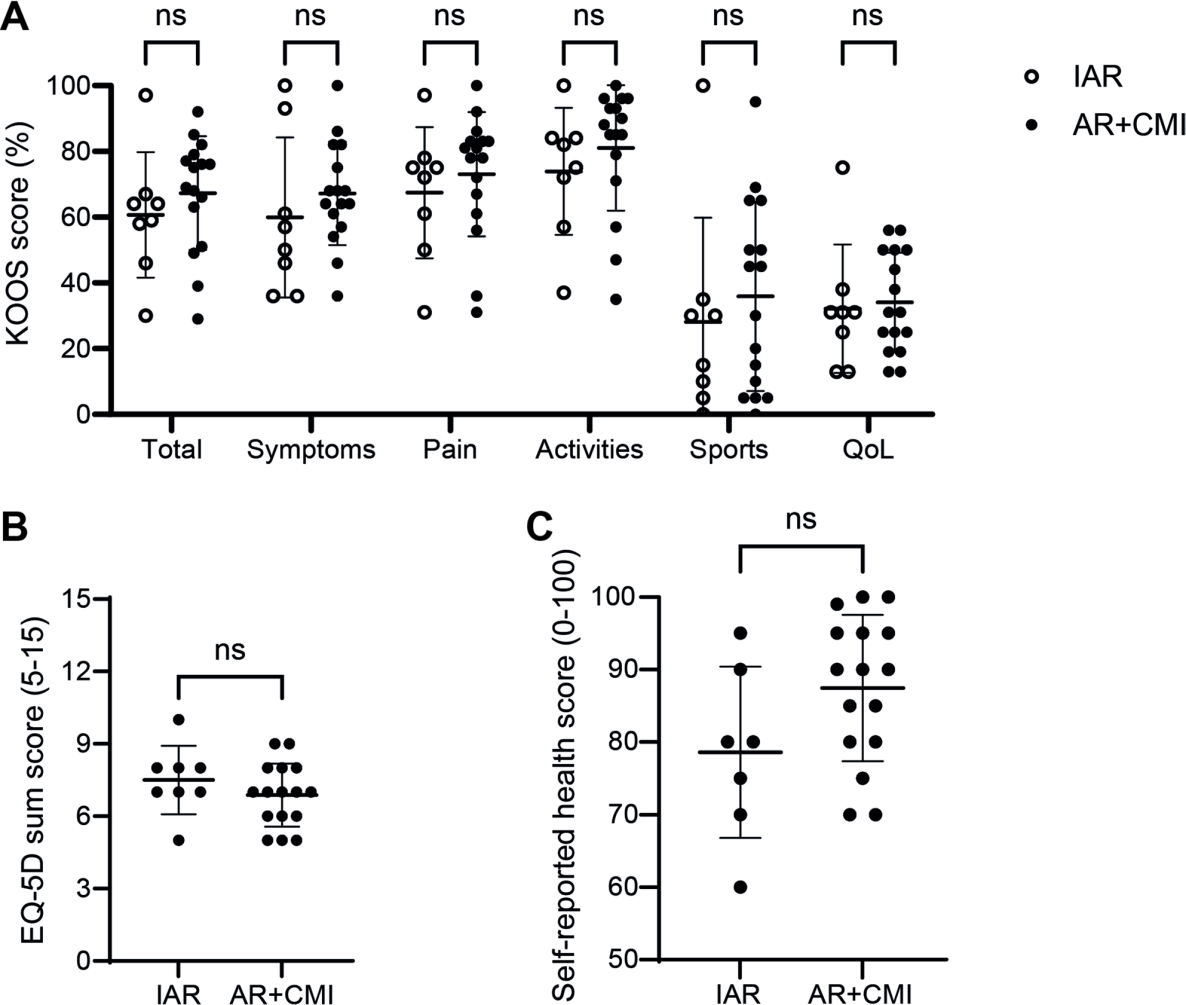
Patients with concurrent meniscal injury do not show worse PROMs at time of ACL reconstruction. **A** Total KOOS score and scores of the individual KOOS subcategories showed a lot of variation between patients, but no differences between the IAR and AR + CMI groups. **B** The EQ-5D sum score showed no significant difference between the IAR and AR + CMI groups. **C** Patients provided a self-reported health score between 0–100 which showed no significant differences between the IAR and AR + CMI groups. KOOS and EQ-5D scores: *n* = 24 patients (IAR: *n* = 8, AR + CMI: *n* = 16); Self-reported health score: *n* = 23 patients (IAR: *n* = 7, AR + CMI: *n* = 16). Horizontal and vertical bars represent mean and standard deviation, respectively. KOOS = Knee Injury and Osteoarthritis Outcome Score; QoL = Quality of Life; IAR = isolated ACL rupture; AR + CMI = ACL rupture + concurrent meniscal injury; ns = not significant

To get a better understanding of the patients’ symptoms and complaints and whether this related to the status of the menisci, we looked at scores from the KOOS and EQ-5D questionnaires that patients completed shortly before ACL reconstruction surgery. We observed a high variation between individual patients in all categories of the KOOS questionnaire. However, this patient variation was not related to the presence of an additional meniscus injury (IAR: *n* = 8; AR + CMI: *n* = 16) (Fig. 4A). Furthermore, the EQ-5D sum score, which is a measure for quality of life within five dimensions, did not differ between IAR and AR + CMI (Fig. 4B). Finally, the patients were asked to provide a score on a scale of 0 to 100 to indicate their general health status at that moment. This self-reported health score did not differ significantly between the IAR and AR + CMI groups (Fig. 4C). Altogether, these data indicate that, contrary to our expectations, patients with a concurrent meniscus injury did not experience worse symptoms compared to patients with an isolated ACL rupture at time of ACL reconstruction.

### ACL reconstruction improves PROMs, while concurrent meniscal injury leads to slightly less improvement after two years

**Fig. 5:**
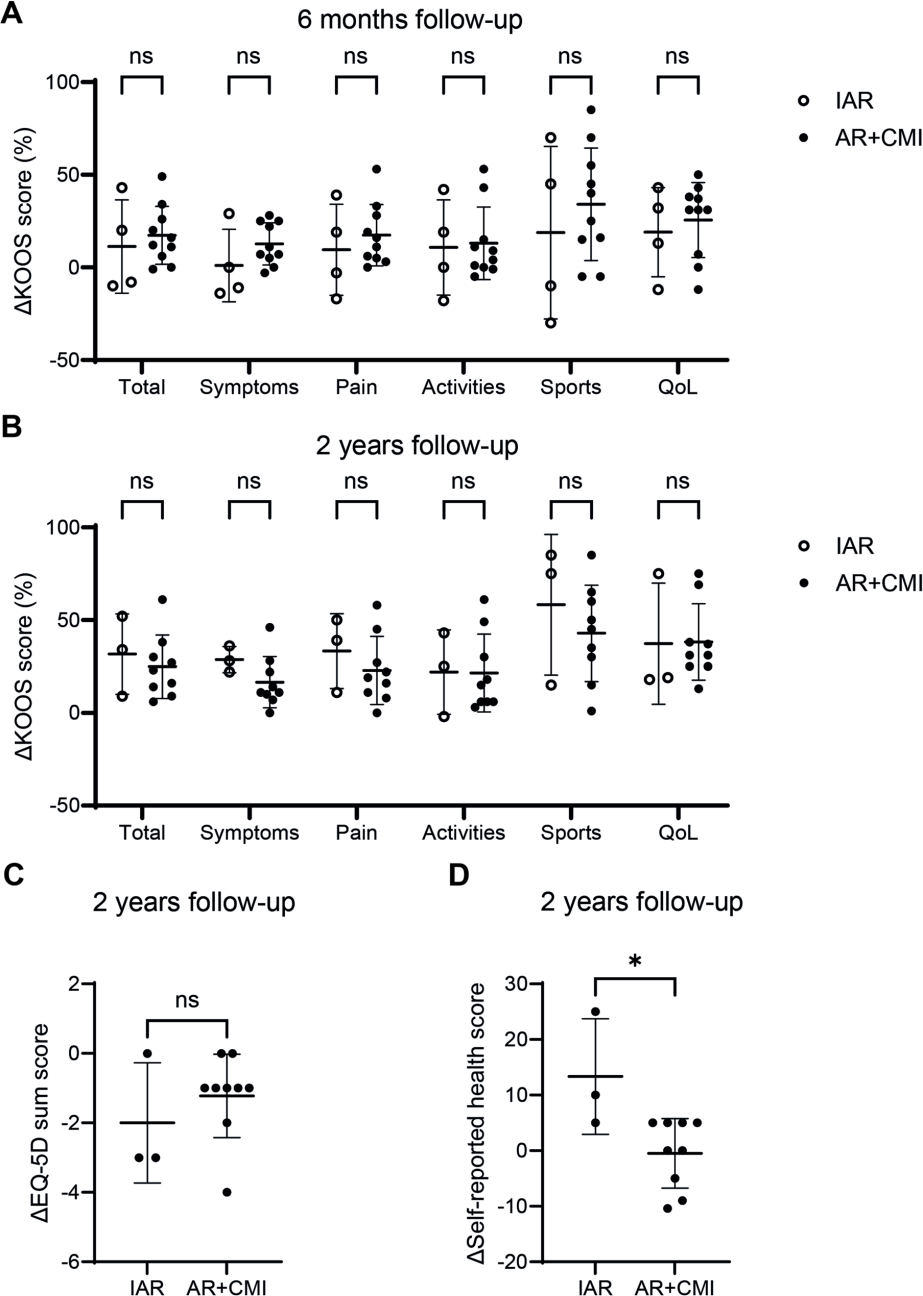
PROMs improve after ACL reconstruction, but patients with concurrent meniscal injury improve slightly less. **A** Total KOOS score and scores of the individual KOOS subcategories 6 months and **B**) 2 years postoperatively showed on average an increase compared to baseline KOOS scores. No differences were found between patients with an isolated ACL rupture and patients with concurrent meniscal injury. **C** EQ-5D sum score 2 years postoperatively showed an average decrease compared to baseline. No differences were found between the IAR and AR + CMI groups. **D** The self-reported health score showed greater improvement two years postoperatively for patients with an isolated ACL rupture compared to patients with concurrent meniscal injury. KOOS 6 months follow-up: *n* = 14 patients (IAR: *n* = 4, AR + CMI: *n* = 10); PROMs 2 years follow-up: *n* = 12 patients (IAR: *n* = 3, AR + CMI: *n* = 9). Horizontal and vertical bars represent mean and standard deviation, respectively. KOOS = Knee Injury and Osteoarthritis Outcome Score; QoL = Quality of Life; IAR = isolated ACL rupture; AR + CMI = ACL rupture + concurrent meniscal injury. *P < 0.05, ns = not significant

As meniscus damage is related to a higher probability for PTOA development, we set out to determine whether concurrent meniscus injury affected the trajectory of PROMS over time, by evaluation of KOOS scores six months postoperatively (IAR: *n* = 4; AR + CMI: *n* = 10) and KOOS and EQ-5D scores two years postoperatively (IAR: *n* = 3; AR + CMI: *n* = 9). We found that after six months the total KOOS score and the scores for the various KOOS sub categories on average improved compared to baseline scores. Although generally an improvement was observed in both groups, we did not find any differences between the IAR and AR + CMI groups at this timepoint (Fig. 5A). Furthermore, at two years follow-up we found an improvement in both KOOS score as well as quality of life measured by the EQ-5D score compared to baseline, but again there were no differences between the IAR and AR + CMI groups (Fig. 5B-C), although the small sample size of follow-up data warrants careful interpretation. Of note, the absolute KOOS and EQ-5D scores did not differ as well between groups at either the six month or two year follow-up moment (Additional File 9 A-C). Interestingly, although the absolute self-reported health score at two years follow-up did not differ between the IAR and AR + CMI groups (Additional File 9D), we observed a greater improvement of the self-reported health score for patients with an isolated ACL rupture compared to patients with concurrent meniscal injury at two years follow-up compared to baseline (Fig. 5D).

### Synovial inflammation varies depending on the time elapsed since ACL rupture

**Fig. 6:**
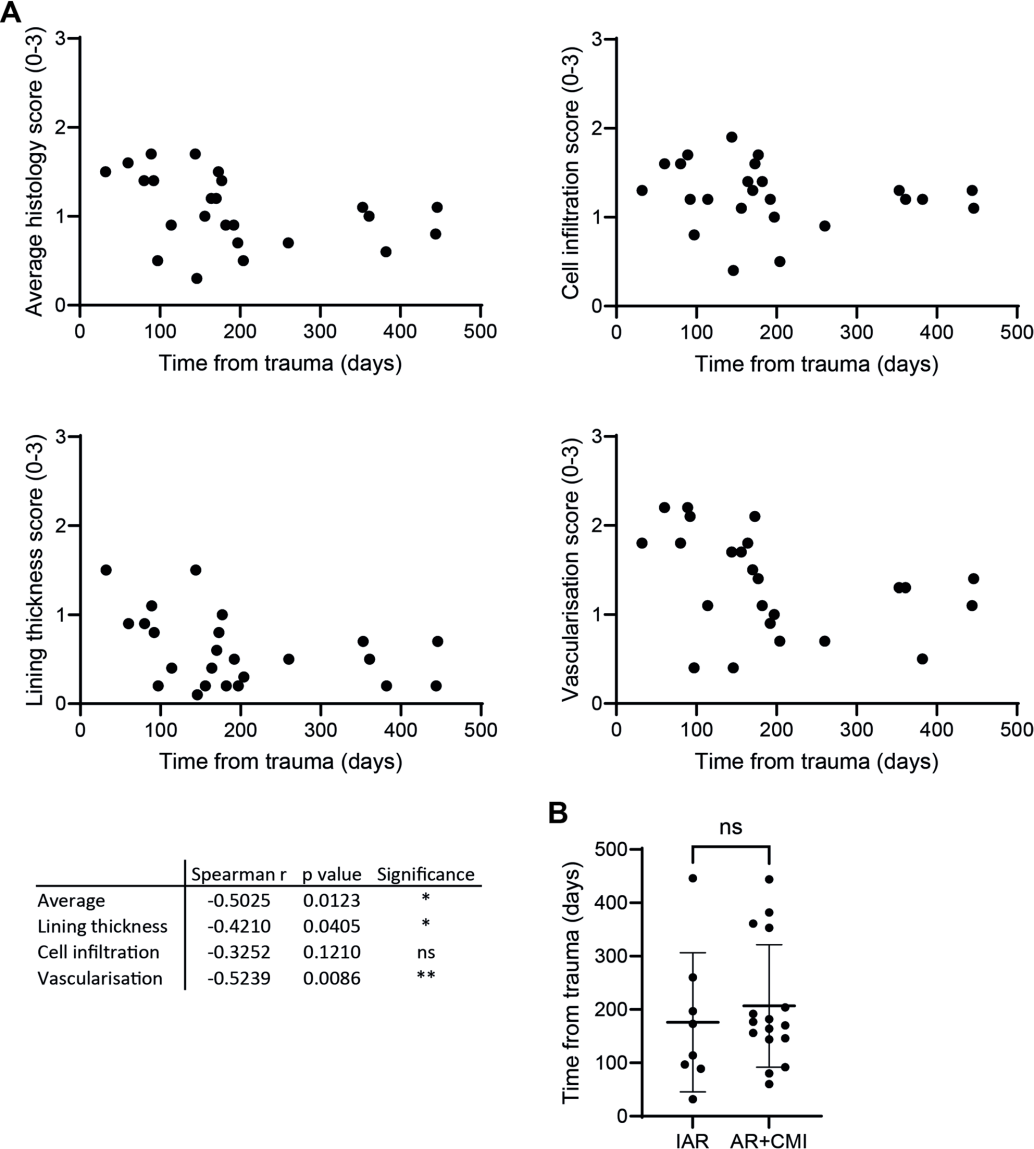
Synovial inflammation varies depending on the time elapsed since ACL rupture. **A** The time from ACL rupture to reconstruction surgery correlated negatively with the average, lining and vascularisation histology scores, indicating higher inflammation earlier after ACL rupture. No correlation was found with the cell infiltration score. **B** Time from ACL rupture to reconstruction surgery did not vary between the IAR and AR + CMI groups, indicating this did not affect our analyses when comparing the two groups. *n* = 24 patients (IAR: *n* = 8, AR + CMI: *n* = 16). Horizontal and vertical bars represent mean and standard deviation, respectively. IAR = isolated ACL rupture; AR + CMI = ACL rupture + concurrent meniscal injury. *P < 0.05, **P < 0.01, ns = not significant

As we did not find any pronounced impact of concurrent meniscal injury on synovitis, we investigated whether there are other factors that might impact the extent of synovial inflammation after ACL rupture. On histology, a negative correlation was found between the time from ACL rupture to reconstruction surgery, and thus sampling of the synovial tissue, and the average, lining and vascularisation scores (Fig. 6A), indicating more inflammation earlier after ACL rupture. No correlation was found with cellular infiltration. Additionally, we did not find correlations between histology scores and BMI or age (Additional File 10 A), or male/female differences (Additional File 10B), which were also not detected in immune cell composition, both local and systemic, or PROMs at baseline and follow-up (data not shown). Subsequently, as we found that the severity of synovitis is related to time after ACL rupture, we looked whether this differed between the IAR and AR + CMI groups, as this might impact the absence of differences that we found when comparing those groups. We found no differences in average time between ACL rupture and reconstruction surgery between the two groups, thus indicating this did not affect our analysis when comparing the two groups (Fig. 6B). Additionally, we investigated whether BMI affected our follow-up data in the AR + CMI patient group, as this can be relevant in the context of altered mechanical loading due to meniscal injury. We did not find any correlations of baseline BMI with Δ PROM scores between baseline and either six months (*n* = 10) or two years (*n* = 9) follow-up (Additional File 11).

## Discussion

In this study, we investigated cellular and molecular inflammatory landscapes locally in the knee joint and systemically in the blood of patients with an ACL rupture. We compared patients with an isolated ACL rupture to patients with concurrent meniscal injury as it is known that concurrent meniscal injury increases the chances of PTOA development. Contrary to our expectations, we did not observe any significant differences in our measured inflammatory and related biological markers between the two groups. This suggests that the higher incidence of PTOA upon concurrent meniscal injury may be driven more by altered joint mechanics, with altered joint loading directly impacting cartilage breakdown, than by distinct systemic or local inflammation at the time of ACL reconstruction surgery. Furthermore, apart from the self-reported health score at two year follow-up, no significant differences in PROMs were observed between the IAR and AR + CMI groups at either baseline or follow-up.

The role of the menisci in preserving joint biomechanics is well established. In a healthy joint, the menisci play an important role in shock absorption, joint stability, joint lubrication, joint movement and load distribution [23, 24]. The mechanical disruption induced by meniscal injury can cause altered or complete loss of these functions and therefore may be a central cause in the development of degenerative changes following ACL rupture. Not only the presence, but also the extent of the meniscal injury or need for surgical intervention seems to be important for pathological outcomes. Previous studies have shown that total meniscectomy is associated with worse long-term outcomes compared to partial meniscectomy [25–27]. The greater the loss of meniscal tissue, the more compromised the biomechanical environment of the knee becomes, leading to altered loading on the cartilage [28]. However, even in the absence of surgical intervention or meniscectomy, the mere presence of a meniscus injury can alter joint loading distribution, potentially contributing to clinical symptoms and cartilage degeneration [29]. These insights have led to a better understanding of the impact of especially total but also partial meniscectomy on joint pathology and caused improved surgical approaches for these patients in the form of meniscal repair [30, 31]. The possibility of performing a repair rather than a (partial) meniscectomy or no surgical intervention is dependent on the location and extent of the tear. The outer area of the meniscus is vascularised, while the inside is not, therefore making the outside area easier to repair as it has the ability to heal [31]. Nowadays, meniscal lesions are more frequently repaired during surgery, generally leading to better outcomes over time [32–34]. These advances in the field of meniscus surgery may have contributed to our observed absence of differences between the IAR and AR + CMI groups at our follow-up timepoints, as better treatment options for meniscus lesions exist nowadays. Additionally, the specific type and location of the meniscal tear may impact joint biomechanics and/or the joint microenvironment. The specific meniscus involved (medial, lateral or both) in addition to different tear patterns, such as longitudinal, radial or complex tears, can have varying effects on the biomechanical function of the meniscus and may therefore differentially influence both joint loading and the local inflammatory landscapes, thereby influencing clinical outcomes following ACL rupture [35, 36]. In the present study, our sample size was relatively small, which limited our ability to perform subgroup analyses based on the specific meniscus involved, tear characteristics or treatment strategy. Future studies with larger cohorts would therefore benefit from examining the impact of tear type, location and treatment approach in more detail. Such analyses could provide further insight into how specific meniscal injury patterns and management strategies influence long-term joint health following ACL injury.

While surgery is often necessary and/or desired by the patients to restore joint stability and function, it represents a secondary trauma to the joint and may introduce additional inflammatory responses [37]. A recent study has found increased pro-inflammatory markers in synovial fluid up to one month post ACL reconstruction surgery [38]. Furthermore, stabilisation of the knee joint by ACL reconstruction does not prevent PTOA development, as studies on conservative therapy and stabilisation surgery show similar incidence of PTOA development [39, 40]. However, untreated instability of the knee joint caused by an ACL tear, especially when the menisci are still intact, can lead to additional joint damage at a later time point, e.g. meniscal injury caused by joint instability [41]. Importantly, in the acute phase after an ACL rupture intra-articular inflammation is typically elevated, which is also reflected in our data. This may negatively affect graft healing and increase the risk of arthrofibrosis if surgery is performed too early [42]. For this reason, delaying reconstruction until the inflammatory response has subsided and range of motion has been restored is often recommended [43]. Taking this all into account, it is important to decide per patient whether surgical intervention is desirable and what the best timing for the surgery is. According to the Dutch guidelines, the following two questions should be asked regarding this timing: (1) How early after knee injury can an ACL reconstruction be safely performed? (2) How long can an ACL reconstruction be safely postponed [44]? Taking this into account, pre-operative rehabilitation might be considered first. Generally, it is advised to perform the ACL reconstruction within five months after ACL rupture. When the ACL is not surgically reconstructed, it is important to use conservative treatment in the form of physical therapy to increase muscle strength and joint stability to minimalize the risk of further injury to the affected joint [45, 46]. It is important to note that the samples analysed in this study were obtained at the time of ACL reconstruction surgery and therefore primarily reflect a subacute to chronic post-injury stage rather than the acute post-traumatic inflammatory phase. Consequently, the absence of significant differences between patients with an isolated ACL rupture and those with concurrent meniscal injury at this timepoint does not exclude the possibility that differences may exist in the very early inflammatory phase after injury. Potential early inflammatory differences may have diminished over time or may be less pronounced at later stages. Furthermore, subtle differences could have remained undetected due to the relatively small sample size.

Although we observed heterogeneity among patients regarding both histology scores as well as immune cell populations in synovium, synovial fluid, and blood, no significant differences were detected between the IAR and AR + CMI groups. Nevertheless, inflammation is thought to be an important contributor to PTOA development [47, 48]. Our flow cytometry data shows a high abundance of macrophages, followed lymphoid cells in the synovium after ACL rupture, but with high inter-patient variability, which seems to be similar to end-stage OA synovium [49, 50]. However, although both patient cohorts show a higher abundance of M2-like over M1-like macrophages in the synovium, it seems that ACL rupture patients has a slightly higher abundance of M1-like macrophages compared to end-stage osteoarthritic synovium, which could be due to an increased inflammatory response shortly after joint trauma [51]. Alternatively, this could be due to the age of the patients, as our patient cohort had a much lower mean age compared to end-stage OA patients, so caution must be taken when making these comparisons. The synovial fluid of our patient cohort shows a distinct pattern compared to synovium, with lymphoid cells being the most abundant immune cells. Whereas the synovial tissue consist of a combination of tissue resident macrophages and infiltrating immune cells, the synovial fluid mainly contains immune cells attracted and migrated into the joint from the circulation, which can explain the observed differences in immune cell composition [52, 53]. A recent study found a similar synovial fluid cell composition following intra-articular ankle fracture, with a high abundance of T cells (72.8% vs. 58.1% lymphoid cells in our data), followed by monocytes (21.2% vs. 23.9% macrophages in our data) [54]. Another study characterised synovial fluid cells in patients with knee osteoarthritis and they found a lower abundance of lymphocytes (44.8%) and a higher abundance of monocytes/macrophages (48.7%) [55]. These differences can be either due to differences in inflammatory stage in an early and late stage of disease, or differences in patient characteristics. Future research should focus on the functions of the specific immune cell subsets in the synovium and synovial fluid and how they can contribute to the local pro-inflammatory environment after joint trauma and eventually the development of PTOA.

Furthermore we looked at synovial gene expression and pathway activation induced by serum and synovial fluid in human chondrocyte-like reporter cell lines. Interesting, our pathway analysis showed the mitogen-activated protein kinase (MAPK) pathway as one of the pathways with an FDR of < 0.05, thereby containing multiple genes with an unadjusted p value of < 0.01 in differential expression between the IAR and AR + CMI groups. Although caution should be taken in drawing strong conclusions from these data as the pathway analysis is based on unadjusted p values, it does hint towards a small difference in inflammatory responses between the two groups. Whether this is relevant for further joint pathology is yet to be determined. Notably, while the AP1 and SRE reporters also involve the MAPK signalling pathway, we did not see different AP1 or SRE activation in our reporter cell lines. An explanation for this might be the difference in input material (synovium vs. synovial fluid and serum) or a difference in sample size between the synovium and synovial fluid, as for not every patient it was possible to extract enough synovial fluid for the reporter cell assays, making the n per group rather small. Although we did not observe significant differences between the IAR and the AR + CMI groups in our reporter cell line data, these data do give an indication of factors present in the blood and synovial fluid of ACL rupture patients. We observed an induction of several pro-inflammatory pathways, like nuclear factor-kappa-B (NF-κB) which can amongst others be activated by interleukin (IL)−1β and SIE which can be activated by IL-6. In contrast, the transforming growth factor (TGF)-β induced reporter cell line, SBE, did not show activation in most patients. These data underline the pro-inflammatory environment that is observed shortly after ACL rupture both in our study as well as other studies [14, 15]. Surprisingly, apart from ontologies concerning extracellular matrix related genes, focal adhesion and the MAPK pathway, several pathway indicating myopathy seemed enriched. Most of the involved genes turned out to be not specific for muscle cells. We hardly found any striated muscle tissue in any of the biopsies, which makes the biological meaning of this finding unsure.

Notably, we did not observe significant differences in patient-reported outcome measures (PROMs) between groups at time of ACL reconstruction surgery. The acute impact of the ACL rupture may overshadow the presence of a concurrent meniscal injury, limiting the sensitivity of PROMs to detect early differences. Similarly, although evidence is arising that early signs of PTOA development can be detected as early as one year after ACL reconstruction surgery [56], our follow-up period may have been too short to detect specifically the impact of concurrent meniscal injury on the process of PTOA development. Therefore, a longer follow-up period of our patient cohort could provide valuable information about possible progression towards PTOA. Furthermore, as meniscal injury may lead to altered mechanical loading, BMI could potentially affect outcomes in patients with concurrent meniscal injury. However, we did not observe correlations between baseline BMI and changes in PROM scores over time in the AR + CMI group, suggesting that BMI was unlikely to be a major determinant of PROM outcomes in this subgroup during the follow-up period. Additionally, the use of pain medication was not systematically recorded or controlled for, as complete information on this variable was not available for all patients. Particularly, differences in type, dosage or duration of pain medication used by patients could have affected perceived pain, function and overall health scores that are reflected by the PROMs. This may have reduced the sensitivity of PROMs to detect potential differences between groups. Within this study, the patient group was rather small, especially regarding the follow-up data, which should therefore be interpreted with caution. Furthermore, we had no knowledge on whether the meniscus injury that was found in these patients occurred at the same time as the ACL rupture, or was either an older or newer injury, which might affect our obtained results.

## Conclusions

In contrast to primary OA, PTOA has a defined onset, offering a window for early intervention. However, there are currently no disease-modifying treatments for OA available and it is largely unknown why certain patients develop OA after joint trauma and others do not. Therefore, research into underlying mechanisms that could lead to PTOA development is important for identification of potential treatment targets. To explore these mechanisms, we compared patients with isolated ACL rupture to those with concurrent meniscal injury. Although we found heterogeneity regarding synovitis among patients at time of ACL reconstruction surgery, the presence of concurrent meniscal injury did not influence cellular and molecular landscapes of inflammation and minimally affected patient reported outcome measures up to two years following ACL reconstruction.

## Supplementary Information

Additional file 1: Flow cytometry staining panels. An overview of the antibodies used for the flow cytometry stainings of the synovium, synovial fluid and blood

Additional file 2: Flow cytometry gating strategy for synovium cells. Synovium from ACL rupture patients was digested to obtain a single-cell suspension which was used for flow cytometric analyses. First, the single, live cells were selected after which we gated for all leukocytes (CD45+). From the leukocyte population, we gated for lymphoid cells (CD3+/CD19+/CD56+/CD117+, low side scatter), mast cells (CD3+/CD19+/CD56+/CD117+, high side scatter), neutrophils (CD15+) and macrophages (CD3-CD19-CD56-CD117-CD68+) From the macrophages, we gated for M1-like macrophages (CD163-HLA-DR+CD86+) and M2-like macrophages (CD163+CD206+)

Additional file 3: Flow cytometry gating strategy for synovial fluid cells. Isolated cells from synovial fluid of ACL rupture patients were used for flow cytometric analyses. First, the single, live cells were selected after which we gated for all leukocytes (CD45+). From the leukocyte population, we gated for lymphoid cells (CD3+/CD19+/CD56+/CD117+, low side scatter), mast cells (CD3+/CD19+/CD56+/CD117+, high side scatter) and myeloid cells (CD11b+). From the myeloid cells, we gated for the CD3-CD19-CD56-CD117- cells and subsequently gated for neutrophils (CD15+) and macrophages (CD15-CD14+). From the macrophages, we gated for M1-like macrophages (CD163-HLA-DR+CD86+) and M2-like macrophages (CD163+CD206+)

Additional file 4: Flow cytometry gating strategy for blood cells. Isolated cells from blood of ACL rupture patients were used for flow cytometric analyses. First, the single, live cells were selected after which we gated for all leukocytes (CD45+). From the leukocyte population, we gated for lymphoid cells (CD11b-CD3+/CD19+/CD56+), monocytes (CD11b+CD14+) and neutrophils (CD11b+CD15+)

Additional File 5: Overview reporter cell lines. An overview of the SW1353 reporter cell lines and their associated signalling pathways

Additional File 6: Reporter validation with known inducers. The SW1353 reporter cell lines were tested for responsiveness by known inducers. NS = not stimulated; IL-1β = interleukin 1β; IL-6 = interleukin 6; TGF-β = transforming growth factor β. FCS = fetal calf serum. ****P < 0.0001.

Additional File 7: Histology scoring system. Histology was scored on a 0-3 scale for lining thickness, cellular infiltration and vascularisation. Black arrows point out representative places indicative for the score. Scale bar = 100 μM

Additional File 8: Pathway analysis in detail. An overview of the eight pathways with an FDR of <0.05 in the pathway analysis. Selected genes with an unadjusted p-value of <0.01 and a log2FoldChange of >0.6 or <-0.6 are marked in red

Additional File 9: Absolute follow-up KOOS and EQ-5D scores. A) Total KOOS score and scores of the individual KOOS subcategories 6 months and B) 2 years postoperatively. No differences were found between patients with an isolated ACL rupture and patients with concurrent meniscal injury. C) EQ-5D sum score and the D) self-reported health score 2 years postoperatively. No differences were found between the IAR and AR+CMI groups. KOOS 6 months follow-up: n=14 patients (IAR: n=4, AR+CMI: n=10); PROMs 2 years follow-up: n=12 patients (IAR: n=3, AR+CMI: n=9). Horizontal and vertical bars represent mean and standard deviation, respectively. KOOS = Knee Injury and Osteoarthritis Outcome Score; QoL = Quality of Life; IAR = isolated ACL rupture; AR+CMI = ACL rupture + concurrent meniscal injury. *P < 0.05, ns = not significant.

Additional File 10: Correlations between histology and demographic data. A) Correlation matrix between histology scores, BMI and age. No correlations were found between the histology scores and BMI or age. B) No differences were found between males and females regarding histology scores. Horizontal and vertical bars represent mean and standard deviation, respectively. BMI = body mass index; ns = not significant

Additional File 11: Correlations between BMI at baseline and Δ PROM scores at six months and two years follow-up in the AR+CMI group

## Data Availability

The datasets used and/or analysed during the current study are available from the corresponding author on reasonable request.

## Abbreviations

ACL: Anterior cruciate ligament
AR + CMI: Anterior cruciate ligament rupture + concurrent meniscal injury
BMI: Body mass index
BSA: Bovine serum albumin
DMEM: Dulbecco’s modified eagle medium
EDTA: Ethylenediaminetetraacetic acid
FACS: Fluorescence-activated cell sorting
FCS: Fetal calf serum
FDR: False discovery rate
H&E: Hematoxylin & Eosin
IAR: Isolated anterior cruciate ligament rupture
IL: Interleukin
KOOS: Knee injury and osteoarthritis outcome score
MAPK: Mitogen-activated protein kinase
METC: Medisch-ethische toetsingscommissie
MMP: Matrix metalloproteinase
NF-κB: Nuclear factor-kappa-B
OA: Osteoarthritis
PBS: Phosphate-buffered saline
PROM: Patient-reported outcome measure
PTOA: Post-traumatic osteoarthritis
RPMI: Roswell Park Memorial Institute
TGF: Transforming growth factor
TM: Thermolysin medium
TPM: Transcripts per million
