# Supplementary material for: Cellular and molecular landscapes of inflammation in anterior cruciate ligament rupture patients are independent on concurrent meniscal injury
Source: Arthritis Res Ther. 2026 Apr 18;28:121. doi: 10.1186/s13075-026-03810-0 (PMC13220405; doi:10.1186/s13075-026-03810-0)
Supplement: Supplementary file 1 — Additional file 1: Flow cytometry staining panels. An overview of the antibodies used for the flow cytometry stainings of the synovium, synovial fluid and blood [file 13075_2026_3810_MOESM1_ESM.pdf]

Additional file 1: Flow cytometry staining panels for synovium, synovial fluid and blood.

Antibody	Fluorochrome	Clone	Supplier	Dilution	Cat#	Synovium	Synovial fluid	Blood
CD3	FITC	HIT3a	BioLegend	1:200	300306	X	X	X
CD19	FITC	HIB19	BioLegend	1:200	302206	X	X	X
CD56	FITC	HCD56	BioLegend	1:50	318304	X	X	X
CD117	FITC	104D2	BioLegend	1:50	313232	X	X	
CD11b	PE/Dazzle594	ICRF44	BioLegend	1:150	301348		X	X
CD206	PE/Cy5	15-2	BioLegend	1:200	321108	X	X	
CD68	PE/Cy7	Y1/82A	BioLegend	1:20	333816	X		
CD14	AlexaFluor700	63D3	BioLegend	1:50	367114		X	X
CD163	BV421	GHI/61	BioLegend	1:100	333612	X	X	
CD45	BV510	HI30	BioLegend	1:200	304036	X	X	X
CD15	BV605	W6D3	BioLegend	1:100	323032	X	X	X
HLA-DR	BV785	L243	BioLegend	1:100	307642	X	X	
CD86	BUV737	2331	BD Biosciences	1:200	612784	X	X	
