# Supplementary material for: Cellular and molecular landscapes of inflammation in anterior cruciate ligament rupture patients are independent on concurrent meniscal injury
Source: Arthritis Res Ther. 2026 Apr 18;28:121. doi: 10.1186/s13075-026-03810-0 (PMC13220405; doi:10.1186/s13075-026-03810-0)
Supplement: Supplementary file 2 — Additional file 2: Flow cytometry gating strategy for synovium cells. Synovium from ACL rupture patients was digested to obtain a single-cell suspension which was used for flow cytometric analyses. First, the single, live cells were selected after which we gated for all leukocytes (CD45+). From the leukocyte population, we gated for lymphoid cells (CD3+/CD19+/CD56+/CD117+, low side scatter), mast cells (CD3+/CD19+/CD56+/CD117+, high side scatter), neutrophils (CD15+) and macrophages (CD3-CD19-CD56-CD117-CD68+) From the macrophages, we gated for M1-like macrophages (CD163-HLA-DR+CD86+) and M2-like macrophages (CD163+CD206+) [file 13075_2026_3810_MOESM2_ESM.pdf]

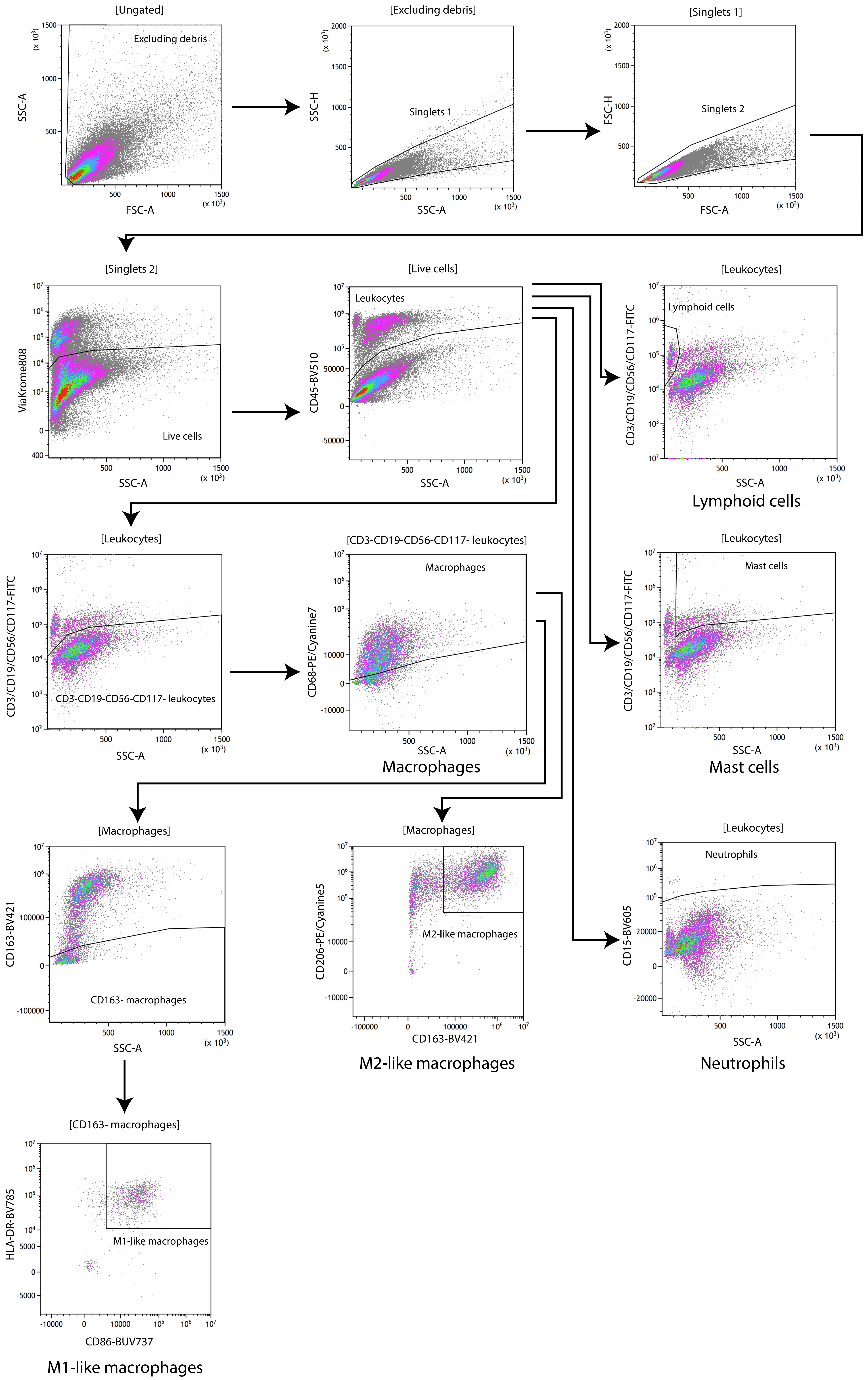

Additional file 2: Flow cytometry gating strategy for synovium cells. Synovium from ACL rupture patients was digested to obtain a single-cell suspension which was used for flow cytometric analyses. First, the single, live cells were selected after which we gated for all leukocytes (CD45+). From the leukocyte population, we gated for lymphoid cells (CD3+/CD19+/CD56+/CD117+, low side scatter), mast cells (CD3+/CD19+/CD56+/CD117+, high side scatter), neutrophils (CD15+) and macrophages (CD3-CD19-CD56-CD117-CD68+) From the macrophages, we gated for M1-like macrophages (CD163-HLA-DR+CD86+) and M2-like macrophages (CD163+CD206+).
