# Supplementary material for: Cellular and molecular landscapes of inflammation in anterior cruciate ligament rupture patients are independent on concurrent meniscal injury
Source: Arthritis Res Ther. 2026 Apr 18;28:121. doi: 10.1186/s13075-026-03810-0 (PMC13220405; doi:10.1186/s13075-026-03810-0)
Supplement: Supplementary file 3 — Additional file 3: Flow cytometry gating strategy for synovial fluid cells. Isolated cells from synovial fluid of ACL rupture patients were used for flow cytometric analyses. First, the single, live cells were selected after which we gated for all leukocytes (CD45+). From the leukocyte population, we gated for lymphoid cells (CD3+/CD19+/CD56+/CD117+, low side scatter), mast cells (CD3+/CD19+/CD56+/CD117+, high side scatter) and myeloid cells (CD11b+). From the myeloid cells, we gated for the CD3-CD19-CD56-CD117- cells and subsequently gated for neutrophils (CD15+) and macrophages (CD15-CD14+). From the macrophages, we gated for M1-like macrophages (CD163-HLA-DR+CD86+) and M2-like macrophages (CD163+CD206+) [file 13075_2026_3810_MOESM3_ESM.pdf]

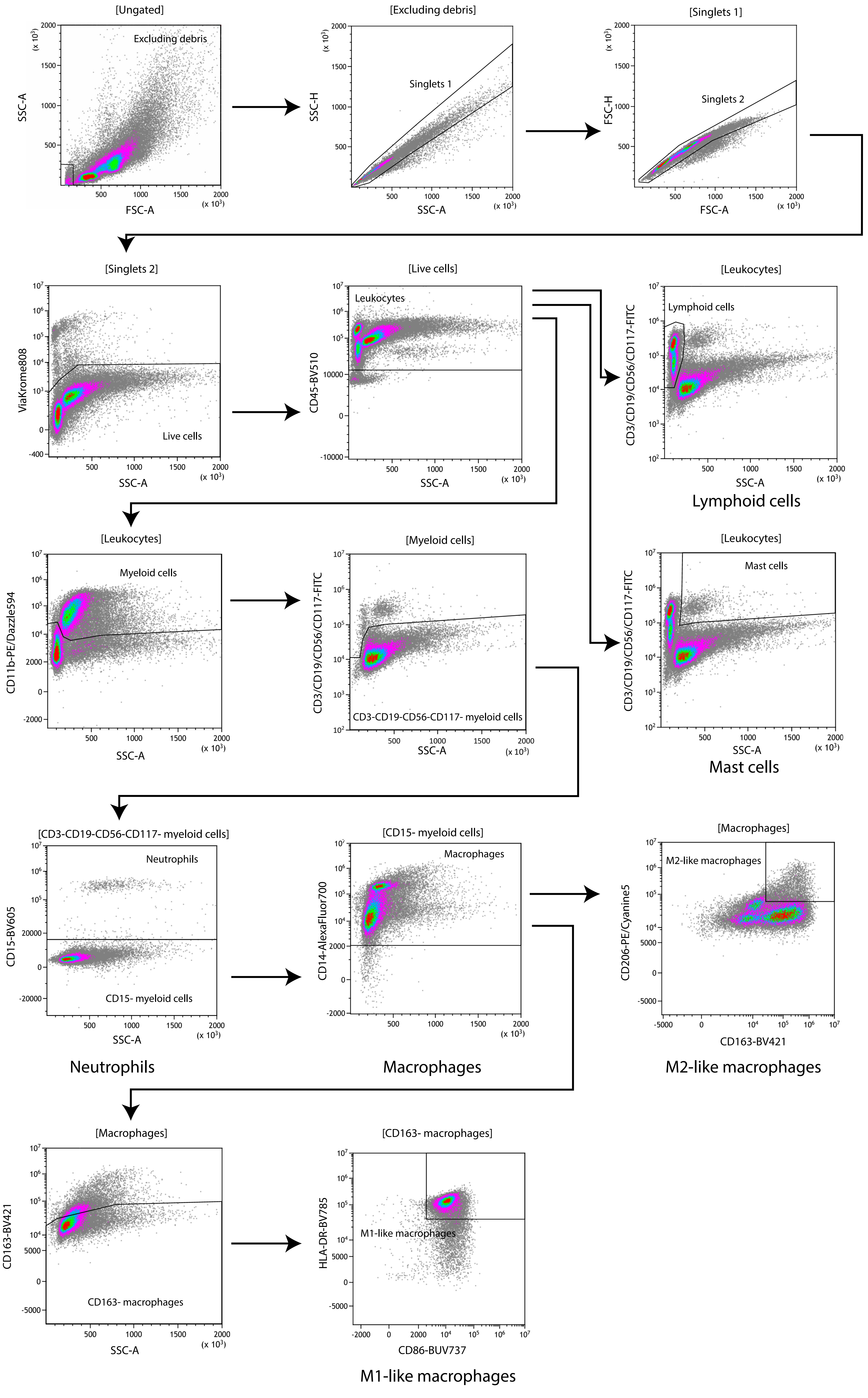

Additional file 3: Flow cytometry gating strategy for synovial fluid cells. Isolated cells from synovial fluid of ACL rupture patients were used for flow cytometric analyses. First, the single, live cells were selected after which we gated for all leukocytes (CD45+). From the leukocyte population, we gated for lymphoid cells (CD3+/CD19+/CD56+/CD117+, low side scatter), mast cells (CD3+/CD19+/CD56+/CD117+, high side scatter) and myeloid cells (CD11b+). From the myeloid cells, we gated for the CD3-CD19-CD56-CD117- cells and subsequently gated for neutrophils (CD15+) and macrophages (CD15-CD14+). From the macrophages, we gated for M1-like macrophages (CD163-HLA-DR+CD86+) and M2-like macrophages (CD163+CD206+).
