# Supplementary material for: Cellular and molecular landscapes of inflammation in anterior cruciate ligament rupture patients are independent on concurrent meniscal injury
Source: Arthritis Res Ther. 2026 Apr 18;28:121. doi: 10.1186/s13075-026-03810-0 (PMC13220405; doi:10.1186/s13075-026-03810-0)
Supplement: Supplementary file 4 — Additional file 4: Flow cytometry gating strategy for blood cells. Isolated cells from blood of ACL rupture patients were used for flow cytometric analyses. First, the single, live cells were selected after which we gated for all leukocytes (CD45+). From the leukocyte population, we gated for lymphoid cells (CD11b-CD3+/CD19+/CD56+), monocytes (CD11b+CD14+) and neutrophils (CD11b+CD15+) [file 13075_2026_3810_MOESM4_ESM.pdf]

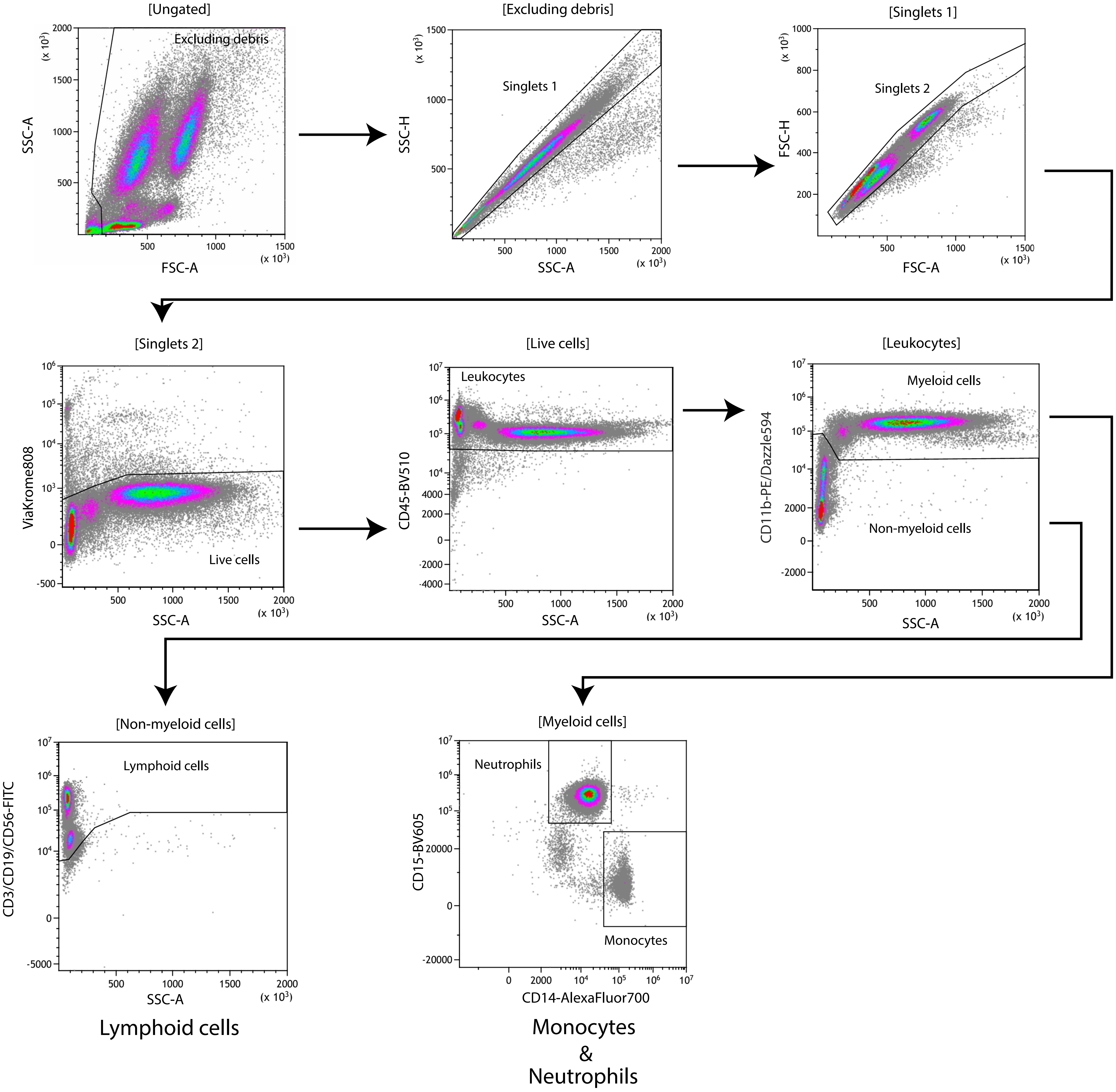

Additional file 4: Flow cytometry gating strategy for blood cells. Isolated cells from blood of ACL rupture patients were used for flow cytometric analyses. First, the single, live cells were selected after which we gated for all leukocytes (CD45+). From the leukocyte population, we gated for lymphoid cells (CD11b-CD3+/CD19+/CD56+), monocytes (CD11b+CD14+) and neutrophils (CD11b+CD15+).
