# Supplementary material for: Cellular and molecular landscapes of inflammation in anterior cruciate ligament rupture patients are independent on concurrent meniscal injury
Source: Arthritis Res Ther. 2026 Apr 18;28:121. doi: 10.1186/s13075-026-03810-0 (PMC13220405; doi:10.1186/s13075-026-03810-0)
Supplement: Supplementary file 5 — Additional File 5: Overview reporter cell lines. An overview of the SW1353 reporter cell lines and their associated signalling pathways [file 13075_2026_3810_MOESM5_ESM.pdf]

Additional file 5: Overview of the SW1353 reporter cell lines

Reporter ID	Response element	Signaling pathway
AP1	Activator protein 1 response element	MAPK signaling pathway (JNK1:2)
ARE	Antioxidant response element	Nrf2 signaling pathway
CRE	cAMP response element	cAMP/PKA (Protein Kinase A) pathway
CSL	CBF1, Suppressor of Hairless, Lag-1 response element	Notch signaling pathway
NFAT5	Nuclear factor of activated T-cells 5 response element	Osmotic stress response pathway
NFkB	Nuclear factor κB response element	NF-κB signaling pathway
SBE	SMAD binding element	TGF-β signaling pathway (SMAD2:3)
SIE	Sis-inducible element	SIS-inducible pathway (STAT1:3)
SRE	Serum response element	MAPK signaling pathway (ERK1)
SRF	Serum response factor response element	RhoA signaling
