# Supplementary material for: Cellular and molecular landscapes of inflammation in anterior cruciate ligament rupture patients are independent on concurrent meniscal injury
Source: Arthritis Res Ther. 2026 Apr 18;28:121. doi: 10.1186/s13075-026-03810-0 (PMC13220405; doi:10.1186/s13075-026-03810-0)
Supplement: Supplementary file 6 — Additional File 6: Reporter validation with known inducers. The SW1353 reporter cell lines were tested for responsiveness by known inducers. NS = not stimulated; IL-1β = interleukin 1β; IL-6 = interleukin 6; TGF-β = transforming growth factor β. FCS = fetal calf serum. ****P < 0.0001. [file 13075_2026_3810_MOESM6_ESM.pdf]

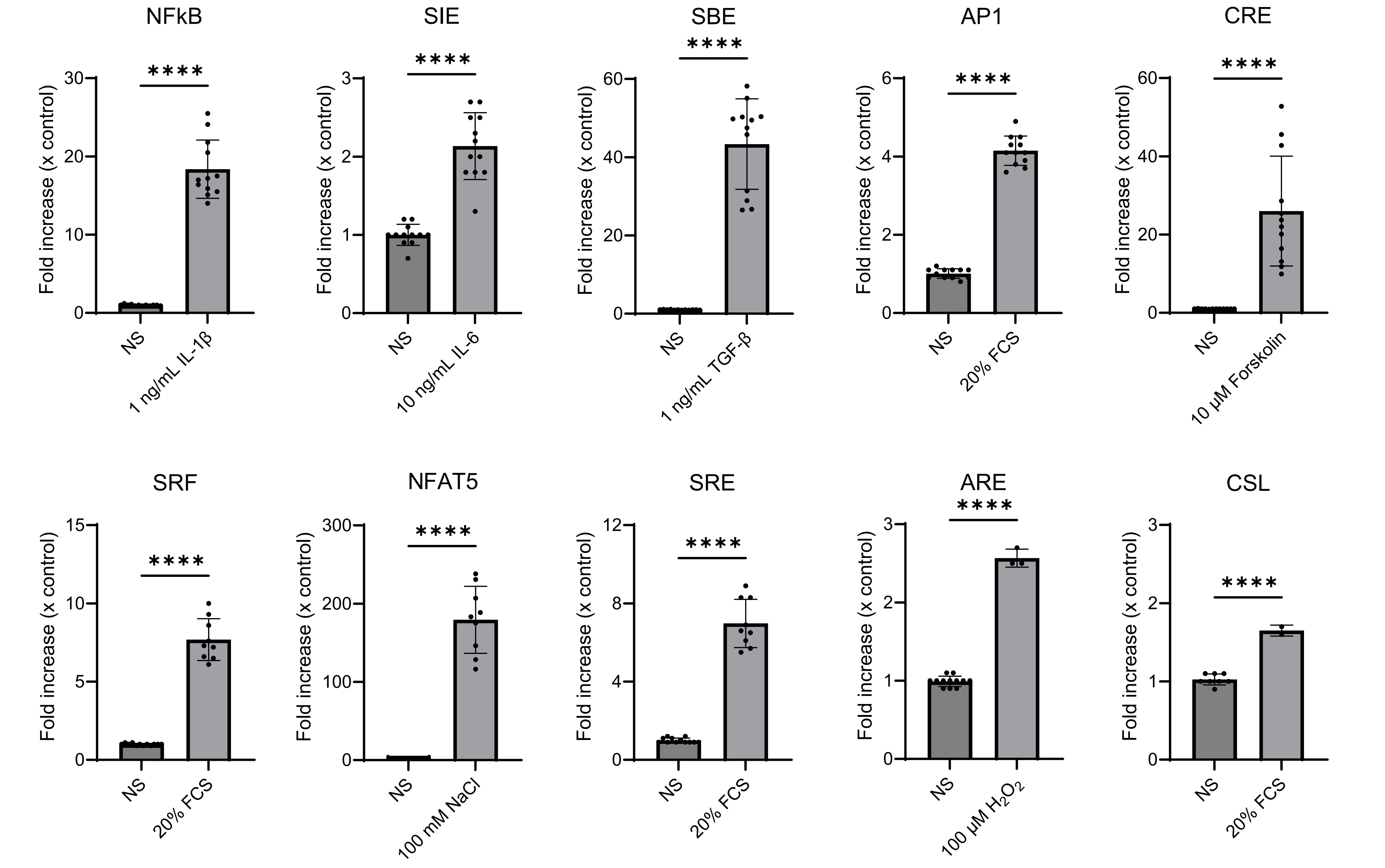

Additional file 6: Reporter validation with known inducers. The SW1353 reporter cell lines were tested for responsiveness by known inducers. Horizontal and vertical bars represent mean and standard deviation, respectively. NS = non-stimulated; IL-1β = interleukin 1β; IL-6 = interleukin 6; TGF-β = transforming growth factor β. FCS = fetal calf serum. ****P < 0.0001.
