# Supplementary material for: Cellular and molecular landscapes of inflammation in anterior cruciate ligament rupture patients are independent on concurrent meniscal injury
Source: Arthritis Res Ther. 2026 Apr 18;28:121. doi: 10.1186/s13075-026-03810-0 (PMC13220405; doi:10.1186/s13075-026-03810-0)
Supplement: Supplementary file 7 — Additional File 7: Histology scoring system. Histology was scored on a 0-3 scale for lining thickness, cellular infiltration and vascularisation. Black arrows point out representative places indicative for the score. Scale bar = 100 μM [file 13075_2026_3810_MOESM7_ESM.pdf]

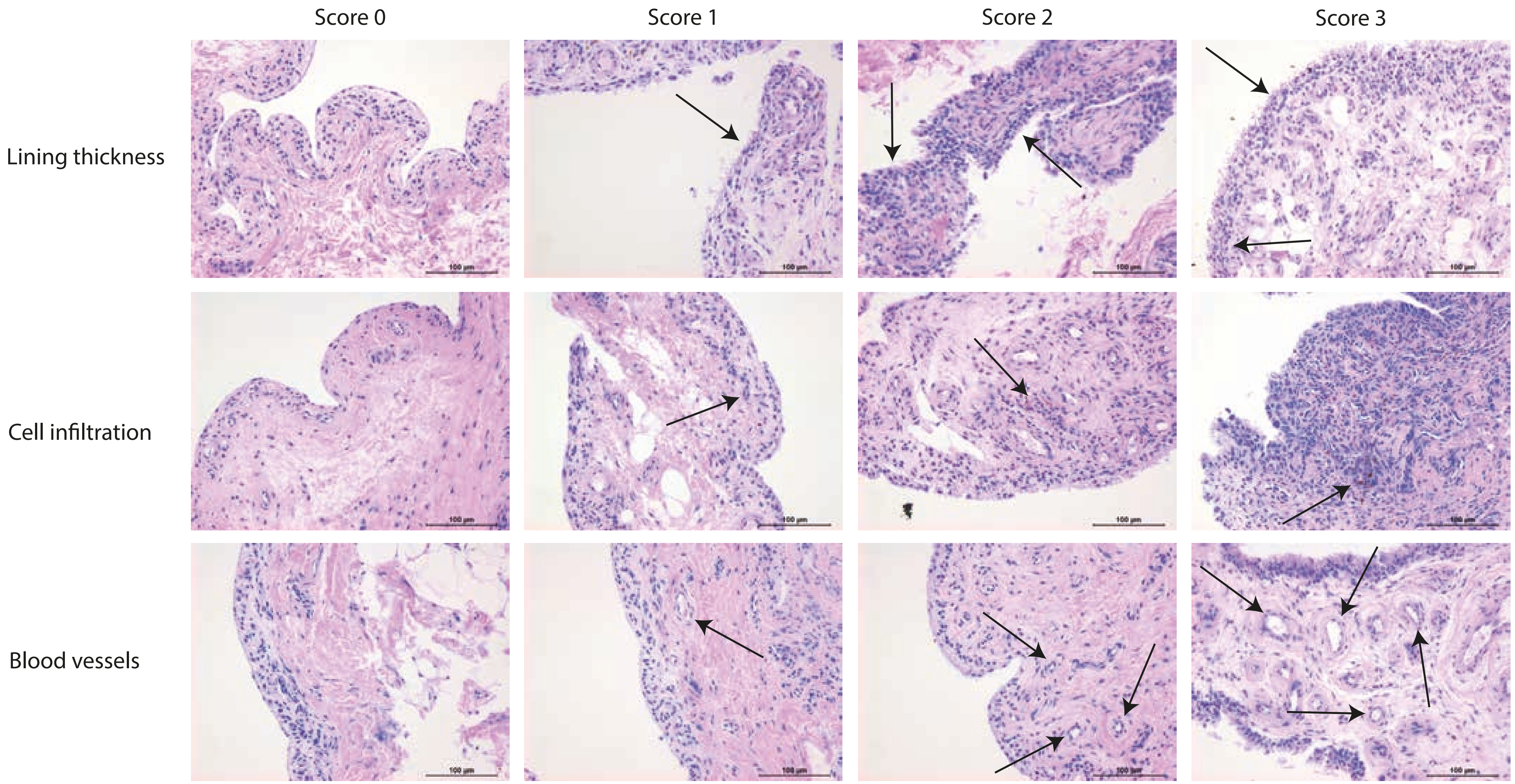

Additional file 7: Histology scoring system. Histology was scored on a 0-3 scale for lining thickness, cellular infiltration and vascularisation. Black arrows point out representative places indicative for the score. Scale bar = 100 μM.
