# Supplementary material for: Cellular and molecular landscapes of inflammation in anterior cruciate ligament rupture patients are independent on concurrent meniscal injury
Source: Arthritis Res Ther. 2026 Apr 18;28:121. doi: 10.1186/s13075-026-03810-0 (PMC13220405; doi:10.1186/s13075-026-03810-0)
Supplement: Supplementary file 8 — Additional File 8: Pathway analysis in detail. An overview of the eight pathways with an FDR of <0.05 in the pathway analysis. Selected genes with an unadjusted p-value of <0.01 and a log2FoldChange of >0.6 or <-0.6 are marked in red [file 13075_2026_3810_MOESM8_ESM.pdf]

Additional File 8: Pathway analysis in detail. An overview of the eight pathways with an FDR of <0.05 in the pathway analysis. Selected genes with an unadjusted p-value of <0.01 and a log2FoldChange of >0.6 or <-0.6 are marked in red.

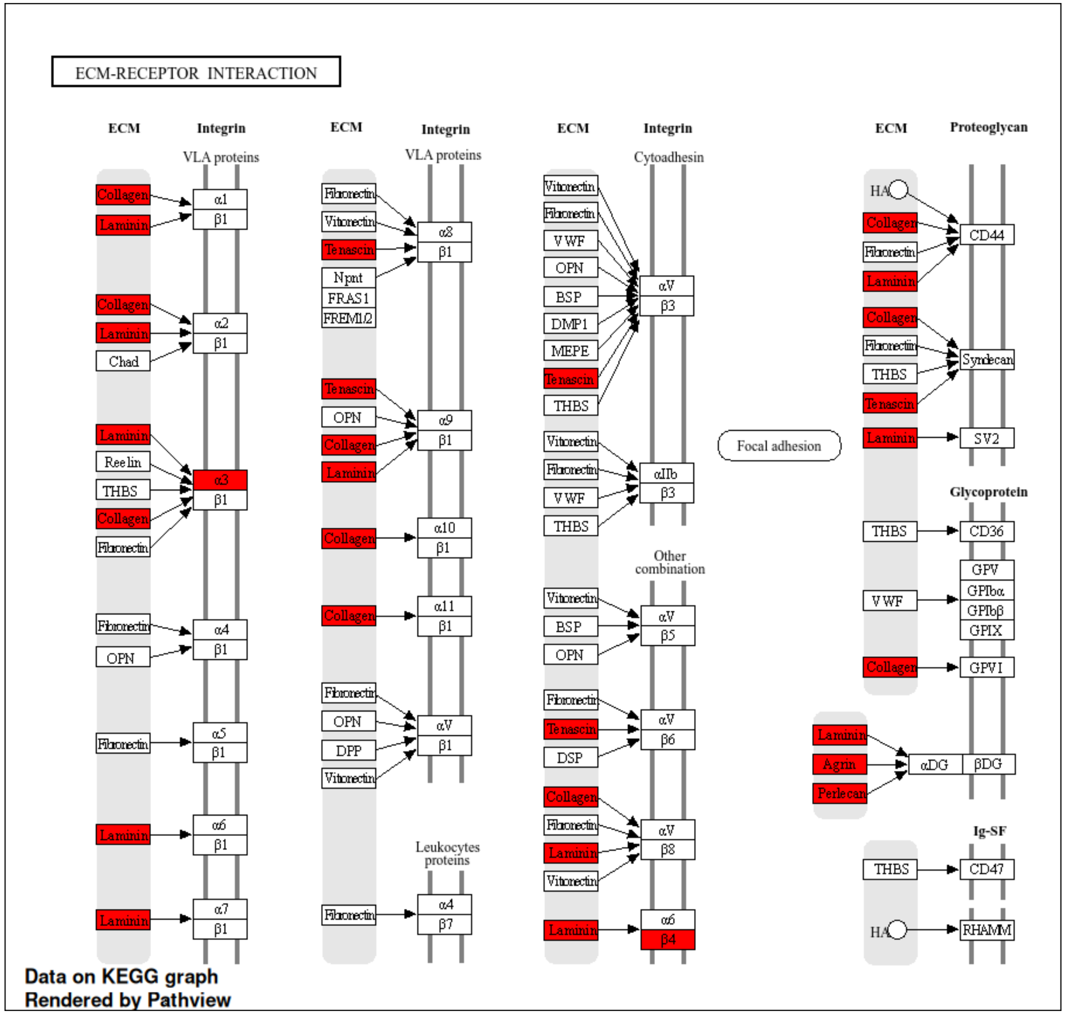

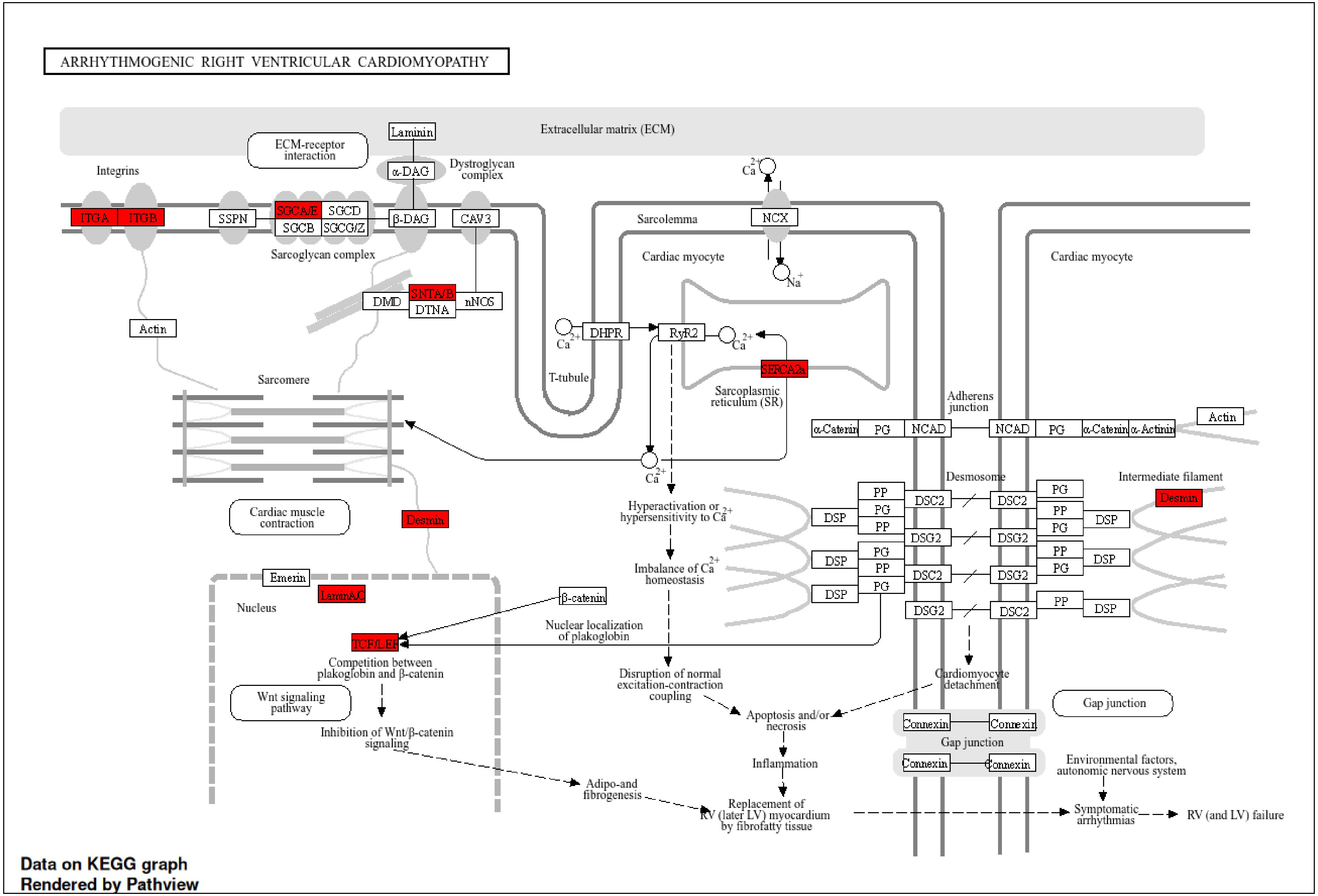
ARRHYTHMOGENIC RIGHT VENTRICULAR CARDIOMYOPATHY
Extracellular matrix (ECM)
Laminin
ECM-receptor interaction
α-DAG
Dystroglycan complex
Integrins
ITGA
ITGB
SSPN
SGCA/E
SGCD
SGCB
SGCG/Z
β-DAG
CAV3
Sarcoglycan complex
DMD
SNTA/B
DTNA
nNOS
Actin
Sarcomere
Cardiac muscle contraction
Desmin
Emerin
LaminA/C
Nucleus
TCF/LEF
Competition between plakoglobin and β-catenin
Wnt signaling pathway
Inhibition of Wnt/β-catenin signaling
β-catenin
Nuclear localization of plakoglobin
Sarcolemma
Cardiac myocyte
NCX
Ca2+
Na+
DHPR
RyR2
SERCA2a
T-tubule
Sarcoplasmic reticulum (SR)
Hyperactivation or hypersensitivity to Ca2+
Imbalance of Ca2+ homeostasis
Disruption of normal excitation-contraction coupling
Apoptosis and/or necrosis
Inflammation
Adipo-and fibrogenesis
Replacement of RV (later LV) myocardium by fibrofatty tissue
Adherens junction
α-Catenin
PG
NCAD
α-Actinin
Desmosome
Intermediate filament
PP
DSP
DSC2
DSG2
Cardiomyocyte detachment
Connexin
Gap junction
Environmental factors, autonomic nervous system
Symptomatic arrhythmias
RV (and LV) failure
Data on KEGG graph
Rendered by Pathview

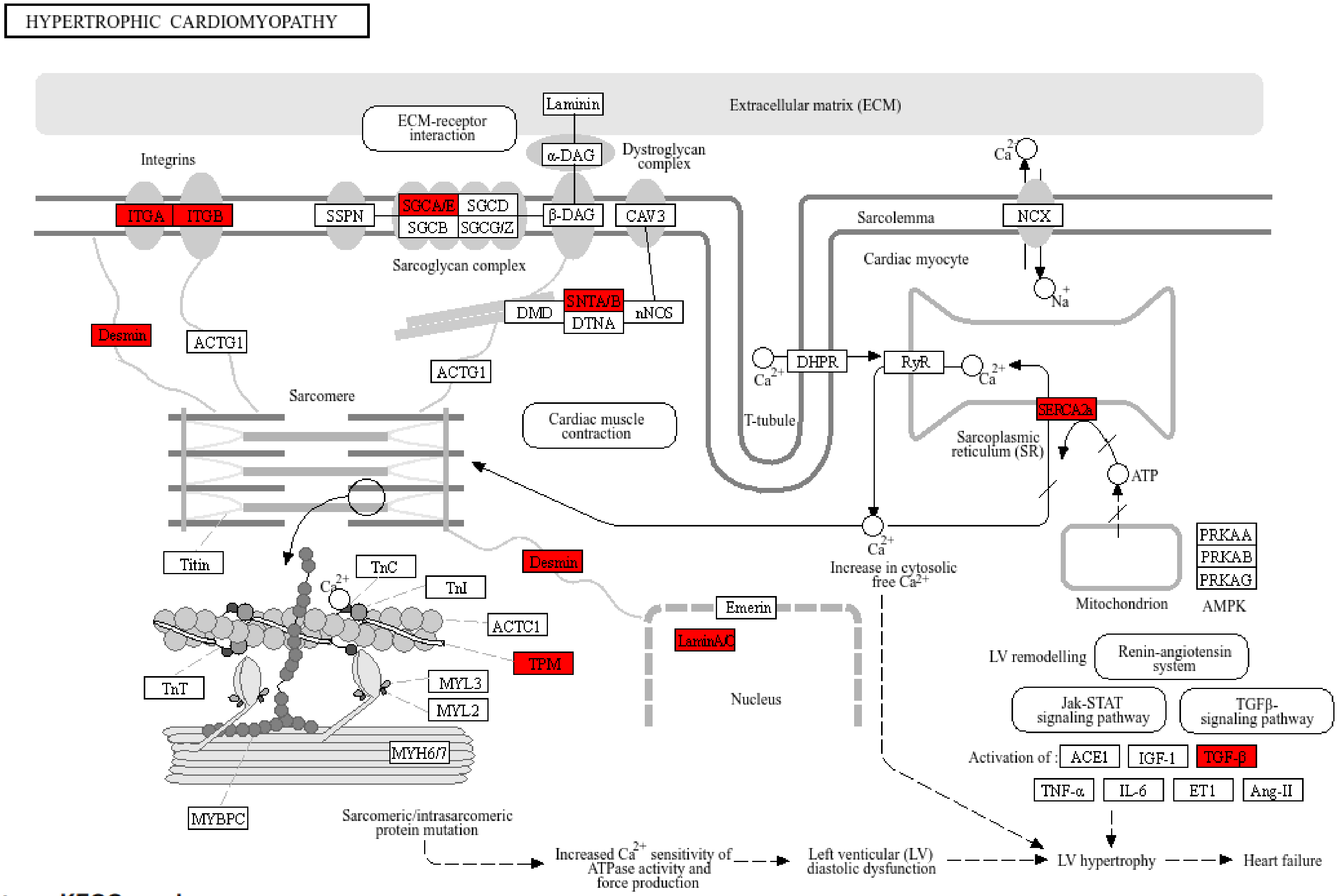

Data on KEGG graph
Rendered by Pathview

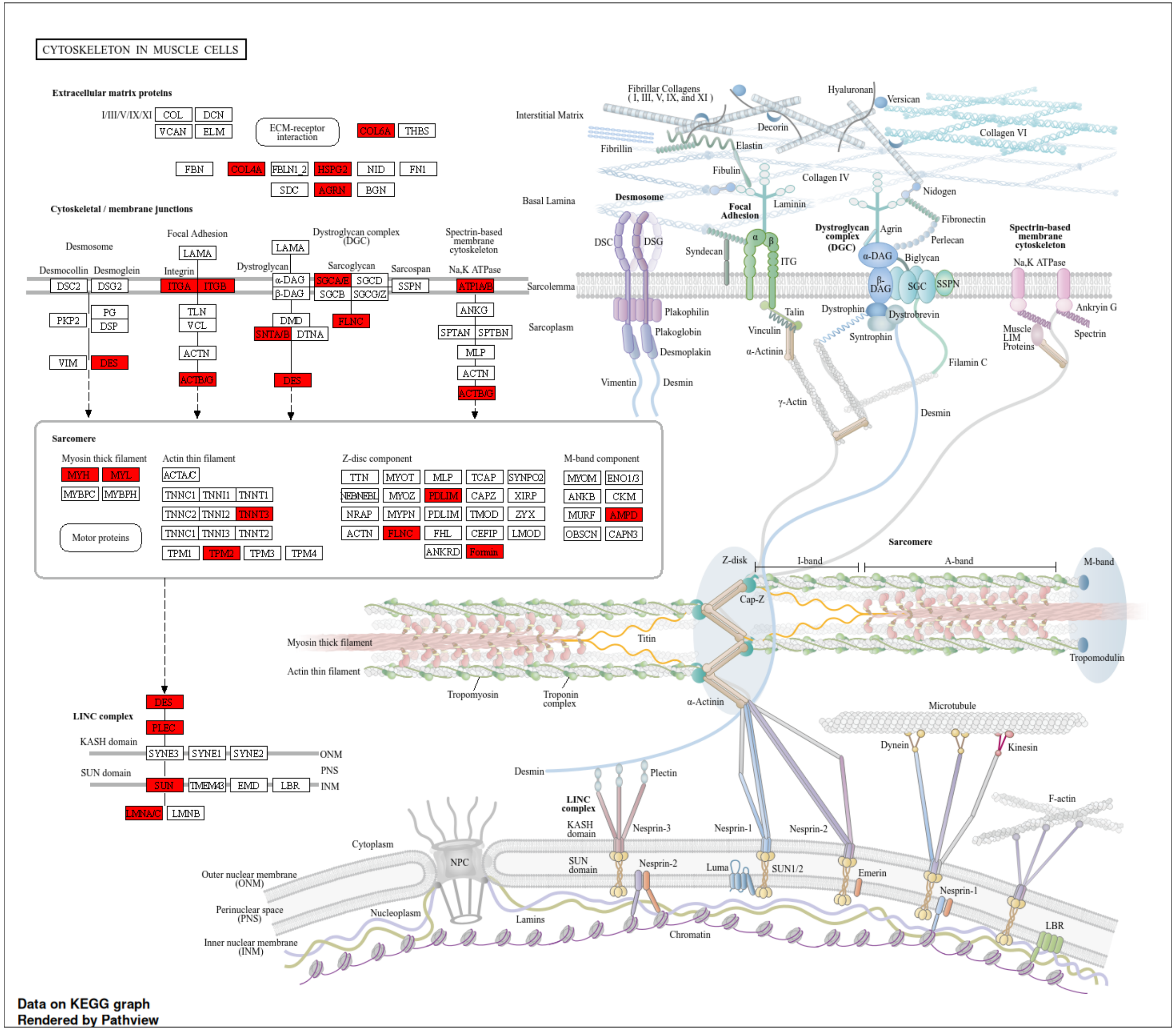

CYTOSKELETON IN MUSCLE CELLS
Extracellular matrix proteins
I/III/V/IX/XI
COL
DCN
VCAN
ELM
ECM-receptor interaction
COL6A
THBS
FBN
COL4A
FBLN1_2
HSPG2
NID
FN1
SDC
AGRN
BGN
Cytoskeletal / membrane junctions
Desmosome
Focal Adhesion
Dystroglycan complex (DGC)
Spectrin-based membrane cytoskeleton
Desmocollin
Desmoglein
Integrin
Dystroglycan
Sarcoglycan
Sarcospan
Na,K ATPase
LAMA
DSC2
DSG2
ITGA
ITGB
α-DAG
β-DAG
SGCA/E
SGCD
SGCB
SGCG/Z
SSPN
ATP1A/B
PKP2
PG
DSP
TLN
VCL
DMD
SNTA/B
DTNA
FLNC
ANKG
SPTAN
SPTBN
VIM
DES
ACTN
ACTB/G
MLP
Sarcolemma
Sarcoplasm
Interstitial Matrix
Basal Lamina
Sarcomere
Myosin thick filament
Actin thin filament
Z-disc component
M-band component
MYH
MYL
MYBPC
MYBPH
ACTA/C
TNNC1
TNNI1
TNNT1
TNNC2
TNNI2
TNNT3
TNNI3
TNNT2
TPM1
TPM2
TPM3
TPM4
Motor proteins
TTN
MYOT
TCAP
SYNPO2
NEB/NEBL
MYOZ
PDLIM
CAPZ
XIRP
NRAP
MYPN
TMOD
ZYX
FLNC
FHL
CEFIP
LMOD
ANKRD
Formin
MYOM
ENO1/3
ANKB
CKM
MURF
AMPD
OBSCN
CAPN3
LINC complex
KASH domain
SUN domain
PLEC
SYNE3
SYNE1
SYNE2
ONM
PNS
INM
SUN
TMEM43
EMD
LBR
LMNA/C
LMNB
Fibrillar Collagens (I, III, V, IX, and XI)
Hyaluronan
Versican
Decorin
Collagen VI
Fibrillin
Elastin
Fibulin
Collagen IV
Nidogen
Desmosome
Focal Adhesion
Laminin
Fibronectin
Dystroglycan complex (DGC)
Agrin
Perlecan
Biglycan
DSC
DSG
Syndecan
α
β
ITG
α-DAG
β-DAG
SGC
SSPN
Plakophilin
Plakoglobin
Desmoplakin
Talin
Vinculin
α-Actinin
Dystrophin
Dystrobrevin
Syntrophin
Filamin C
Spectrin-based membrane cytoskeleton
Na,K ATPase
Ankryin G
Spectrin
Muscle LIM Proteins
Vimentin
Desmin
γ-Actin
Desmin
Sarcomere
Z-disk
I-band
A-band
M-band
Cap-Z
Titin
Myosin thick filament
Actin thin filament
Tropomodulin
Tropomyosin
Troponin complex
α-Actinin
Microtubule
Dynein
Kinesin
Desmin
Plectin
F-actin
LINC complex
KASH domain
Nesprin-3
Nesprin-1
Nesprin-2
SUN domain
Nesprin-2
Luma
SUN1/2
Emerin
Nesprin-1
LBR
Cytoplasm
NPC
Outer nuclear membrane (ONM)
Perinuclear space (PNS)
Inner nuclear membrane (INM)
Nucleoplasm
Lamins
Chromatin
Data on KEGG graph
Rendered by Pathview

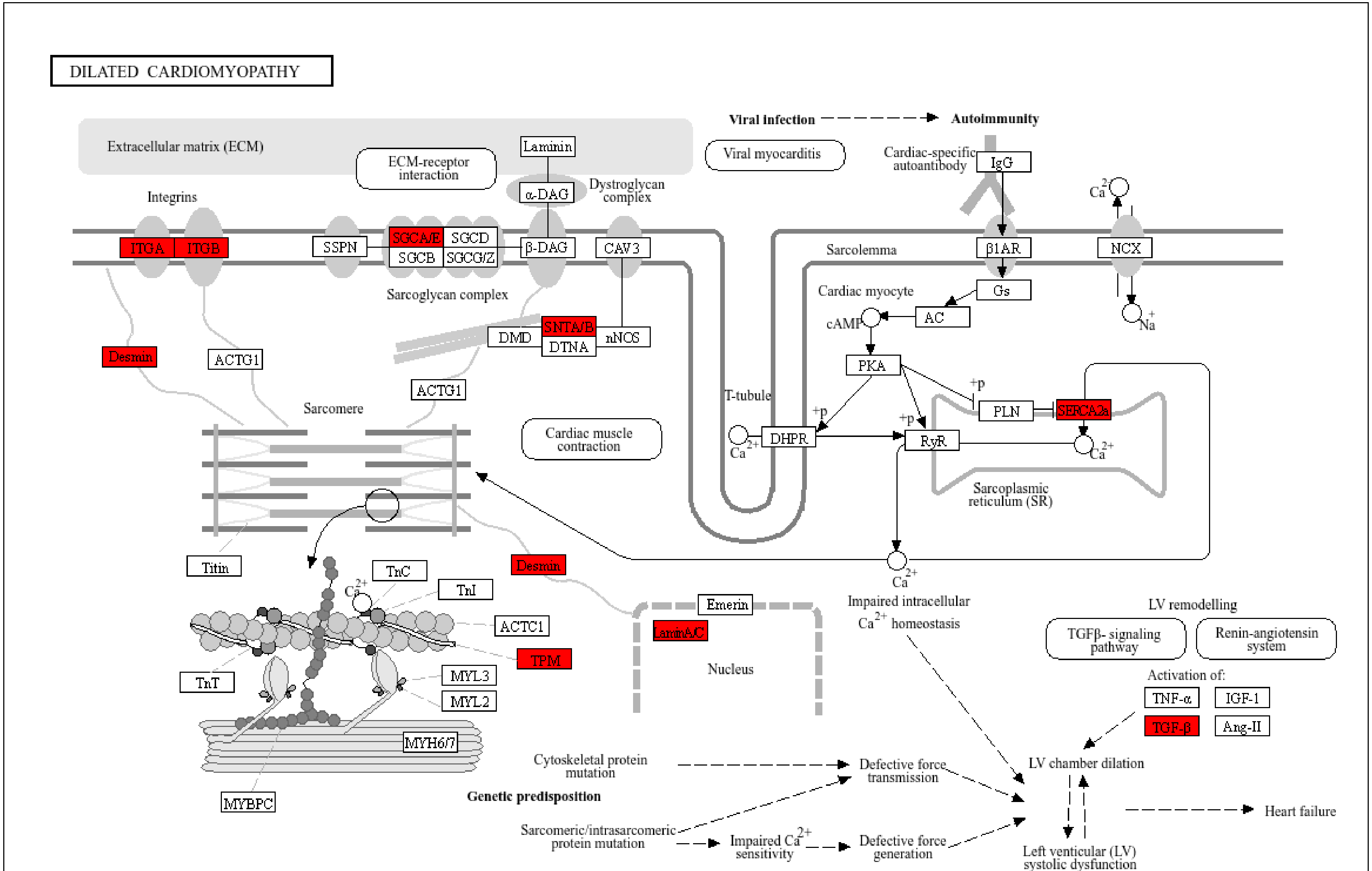

Data on KEGG graph
Rendered by Pathview

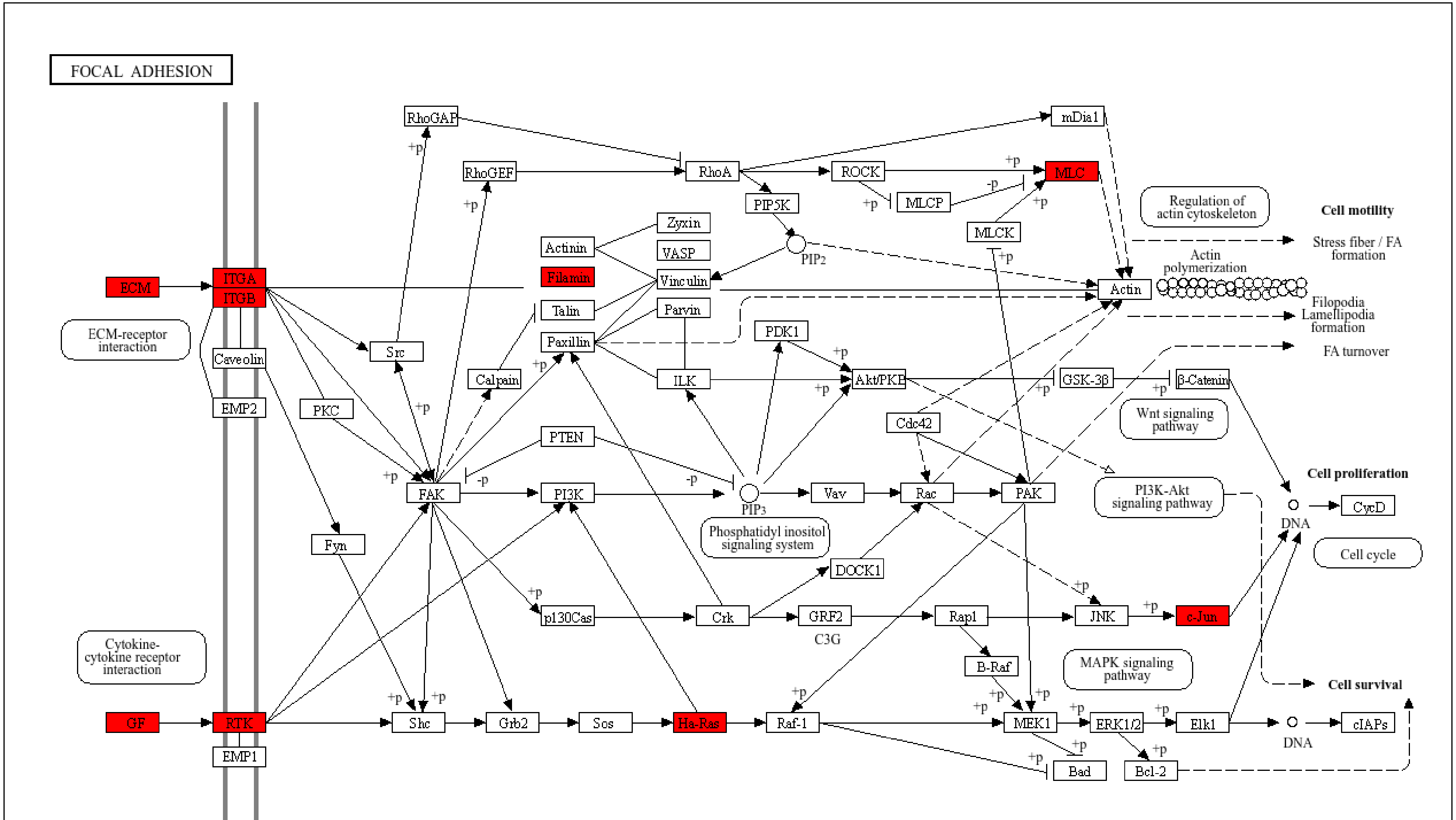

Data on KEGG graph
Rendered by Pathview

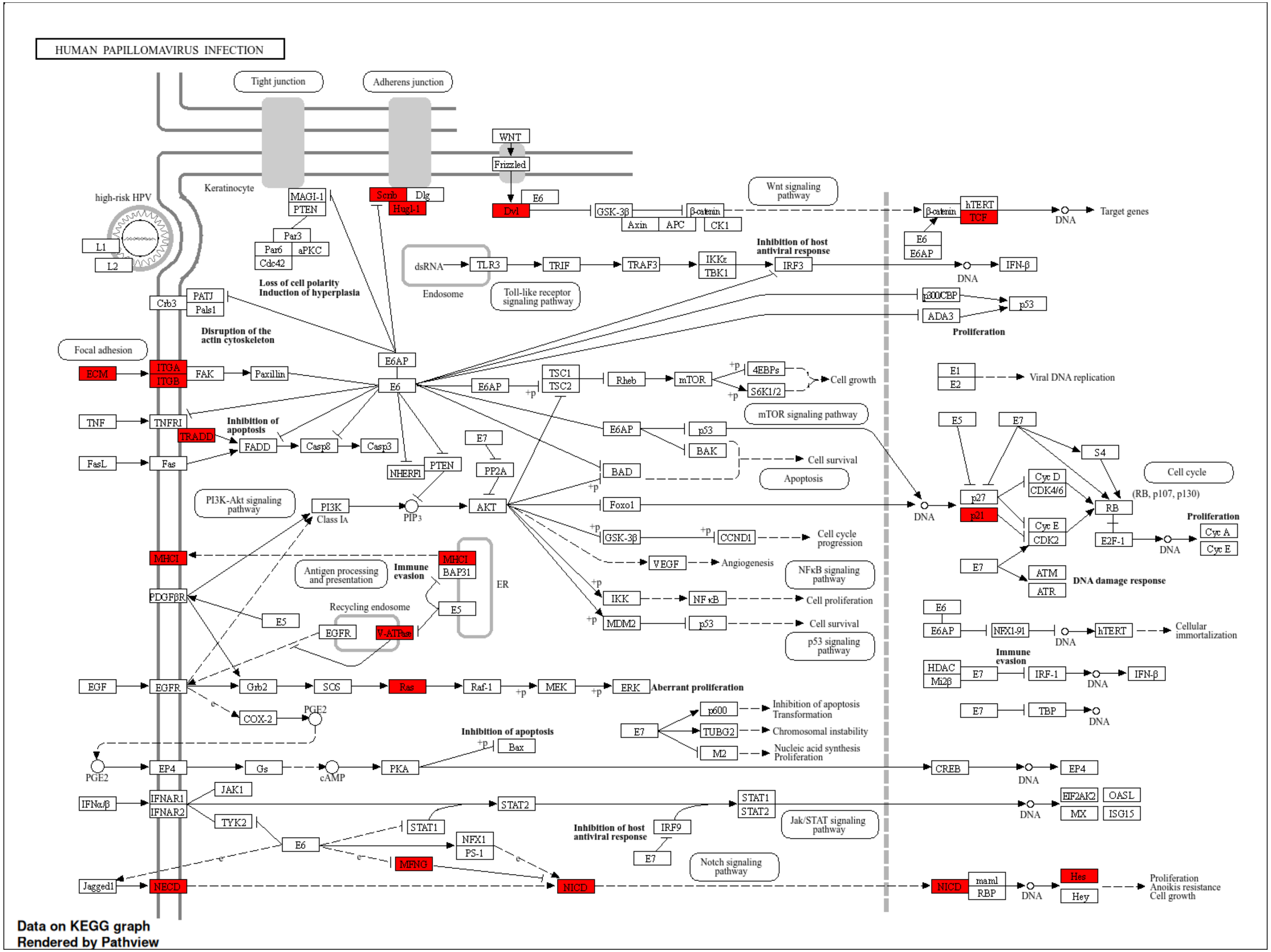
HUMAN PAPILLOMAVIRUS INFECTION
Tight junction
Adherens junction
WNT
Frizzled
Keratinocyte
high-risk HPV
L1
L2
MAGI-1
PTEN
Scrib
Dlg
Hugl-1
Dvl
E6
GSK-3β
β-catenin
Axin
APC
CK1
Wnt signaling pathway
β-catenin
hTERT
TCF
DNA
Target genes
E6
E6AP
Par3
Par6
aPKC
Cdc42
dsRNA
TLR3
TRIF
TRAF3
IKKε
TBK1
IRF3
IFN-β
Inhibition of host antiviral response
Endosome
Toll-like receptor signaling pathway
Loss of cell polarity
Induction of hyperplasia
Crb3
PATJ
Pals1
p300/CBP
ADA3
p53
Proliferation
Disruption of the actin cytoskeleton
Focal adhesion
ECM
ITGA
ITGB
FAK
Paxillin
E6AP
E6
TSC1
TSC2
Rheb
mTOR
4EBPs
S6K1/2
Cell growth
mTOR signaling pathway
E1
E2
Viral DNA replication
TNF
TNFR1
TRADD
Inhibition of apoptosis
FADD
Casp8
Casp3
NHERF1
PTEN
E7
PP2A
BAK
BAD
Cell survival
Apoptosis
E5
E7
S4
Cyc D
CDK4/6
p27
p21
RB
(RB, p107, p130)
Cell cycle
FasL
Fas
PI3K-Akt signaling pathway
PI3K
Class IA
PIP3
AKT
Foxo1
GSK-3β
CCND1
Cell cycle progression
Cyc E
CDK2
E2F-1
Cyc A
Cyc E
Proliferation
VEGF
Angiogenesis
NFκB signaling pathway
MHCI
MHCI
BAP31
Antigen processing and presentation
Immune evasion
ER
PDGFβR
E5
Recycling endosome
E5
EGFR
V-ATPase
IKK
NFκB
Cell proliferation
MDM2
p53
p53 signaling pathway
ATM
ATR
DNA damage response
E6
E6AP
NFX1-91
hTERT
Cellular immortalization
Immune evasion
HDAC
Mi2β
E7
IRF-1
IFN-β
EGF
EGFR
Grb2
SOS
Ras
Raf-1
MEK
ERK
Aberrant proliferation
COX-2
PGE2
p600
Inhibition of apoptosis
Transformation
E7
TUBG2
Chromosomal instability
M2
Nucleic acid synthesis
Proliferation
TBP
Bax
EP4
Gs
cAMP
PKA
CREB
EP4
IFNα/β
IFNAR1
IFNAR2
JAK1
STAT2
STAT1
STAT2
EIF2AK2
OASL
MX
ISG15
TYK2
Jak/STAT signaling pathway
E6
STAT1
NFX1
PS-1
Inhibition of host antiviral response
IRF9
E7
MFNG
Notch signaling pathway
Jagged1
NECD
NICD
NICD
maml
RBP
Hes
Hey
Proliferation
Anoikis resistance
Cell growth
Data on KEGG graph
Rendered by Pathview

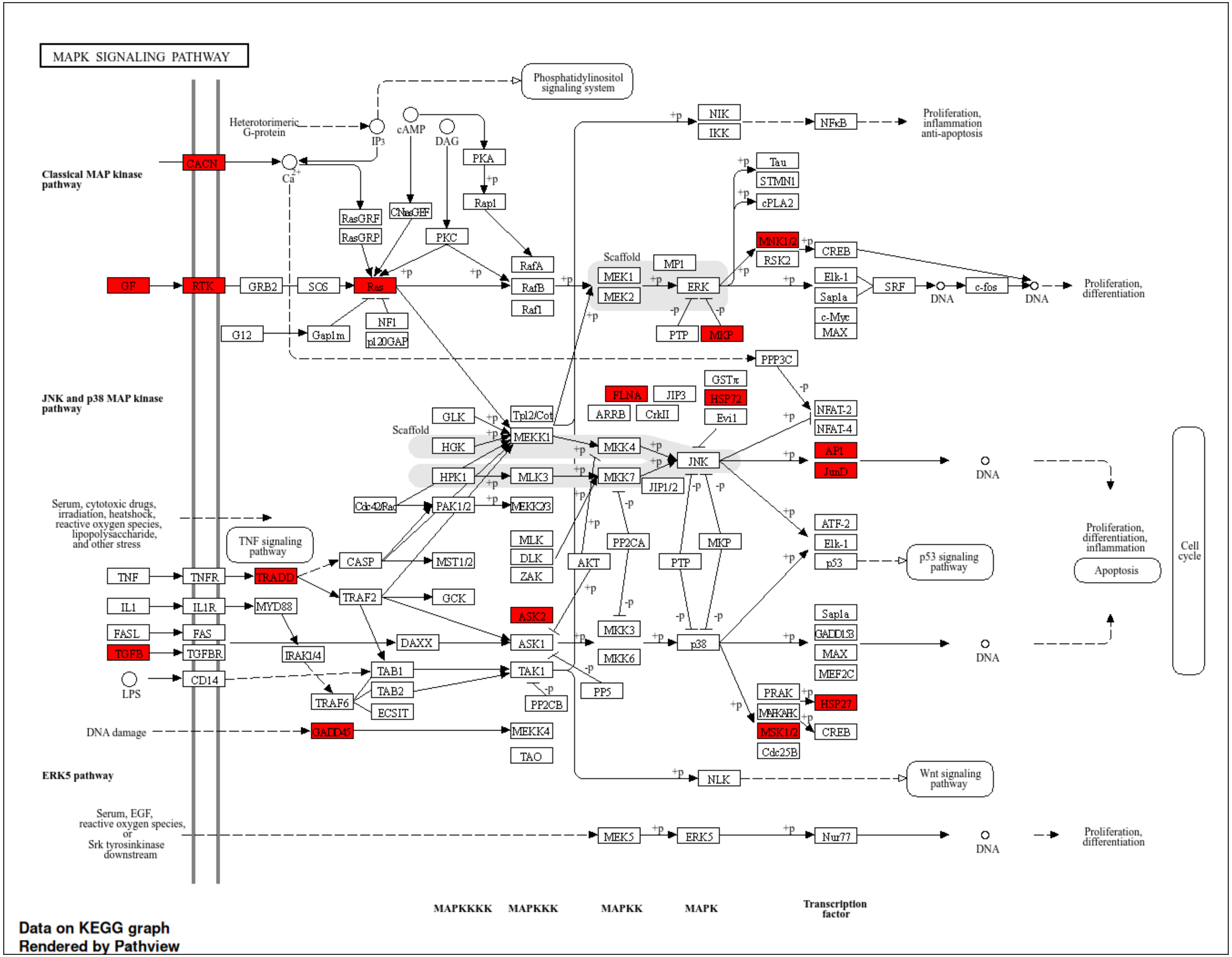
MAPK SIGNALING PATHWAY
Classical MAP kinase pathway
JNK and p38 MAP kinase pathway
ERK5 pathway
Phosphatidylinositol signaling system
Heterotorimeric G-protein
IP3
cAMP
DAG
Ca2+
CACN
PKA
Rap1
RasGRF
RasGRP
CNrasGEF
PKC
GF
RTK
GRB2
SOS
Ras
NF1
p120GAP
G12
Gap1m
RafA
RafB
Raf1
Scaffold
MEK1
MEK2
MP1
ERK
PTP
MKP
NIK
IKK
NFκB
Proliferation, inflammation anti-apoptosis
Tau
STMN1
cPLA2
MNK1/2
RSK2
CREB
Elk-1
Sap1a
SRF
c-fos
c-Myc
MAX
DNA
Proliferation, differentiation
PPP3C
NFAT-2
NFAT-4
FLNA
JIP3
GSTπ
HSP72
Evi1
ARRB
CrkII
GLK
HGK
HPK1
Tpl2/Cot
MEKK1
MLK3
MEKK2/3
MKK4
MKK7
JIP1/2
JNK
AP1
JunD
Cdc42/Rac
PAK1/2
Serum, cytotoxic drugs, irradiation, heatshock, reactive oxygen species, lipopolysaccharide, and other stress
TNF signaling pathway
CASP
MST1/2
MLK
DLK
ZAK
AKT
PP2CA
MKP
PTP
ATF-2
Elk-1
p53
p53 signaling pathway
Proliferation, differentiation, inflammation
Apoptosis
Cell cycle
TNF
TNFR
TRADD
TRAF2
GCK
IL1
IL1R
MYD88
ASK2
FASL
FAS
DAXX
ASK1
MKK3
MKK6
p38
Sap1a
GADD153
MAX
MEF2C
TGFB
TGFBR
IRAK1/4
TAB1
TAK1
TAB2
TRAF6
ECSIT
PP2CB
PP5
PRAK
MAPKAPK
MSK1/2
HSP27
CREB
Cdc25B
LPS
CD14
DNA damage
GADD45
MEKK4
TAO
NLK
Wnt signaling pathway
Serum, EGF, reactive oxygen species, or Srk tyrosinkinase downstream
MEK5
ERK5
Nur77
MAPKKKK
MAPKKK
MAPKK
MAPK
Transcription factor
Data on KEGG graph
Rendered by Pathview
