# Supplementary material for: Cellular and molecular landscapes of inflammation in anterior cruciate ligament rupture patients are independent on concurrent meniscal injury
Source: Arthritis Res Ther. 2026 Apr 18;28:121. doi: 10.1186/s13075-026-03810-0 (PMC13220405; doi:10.1186/s13075-026-03810-0)
Supplement: Supplementary file 9 — Additional File 9: Absolute follow-up KOOS and EQ-5D scores. A) Total KOOS score and scores of the individual KOOS subcategories 6 months and B) 2 years postoperatively. No differences were found between patients with an isolated ACL rupture and patients with concurrent meniscal injury. C) EQ-5D sum score and the D) self-reported health score 2 years postoperatively. No differences were found between the IAR and AR+CMI groups. KOOS 6 months follow-up: n=14 patients (IAR: n=4, AR+CMI: n=10); PROMs 2 years follow-up: n=12 patients (IAR: n=3, AR+CMI: n=9). Horizontal and vertical bars represent mean and standard deviation, respectively. KOOS = Knee Injury and Osteoarthritis Outcome Score; QoL = Quality of Life; IAR = isolated ACL rupture; AR+CMI = ACL rupture + concurrent meniscal injury. *P < 0.05, ns = not significant. [file 13075_2026_3810_MOESM9_ESM.pdf]

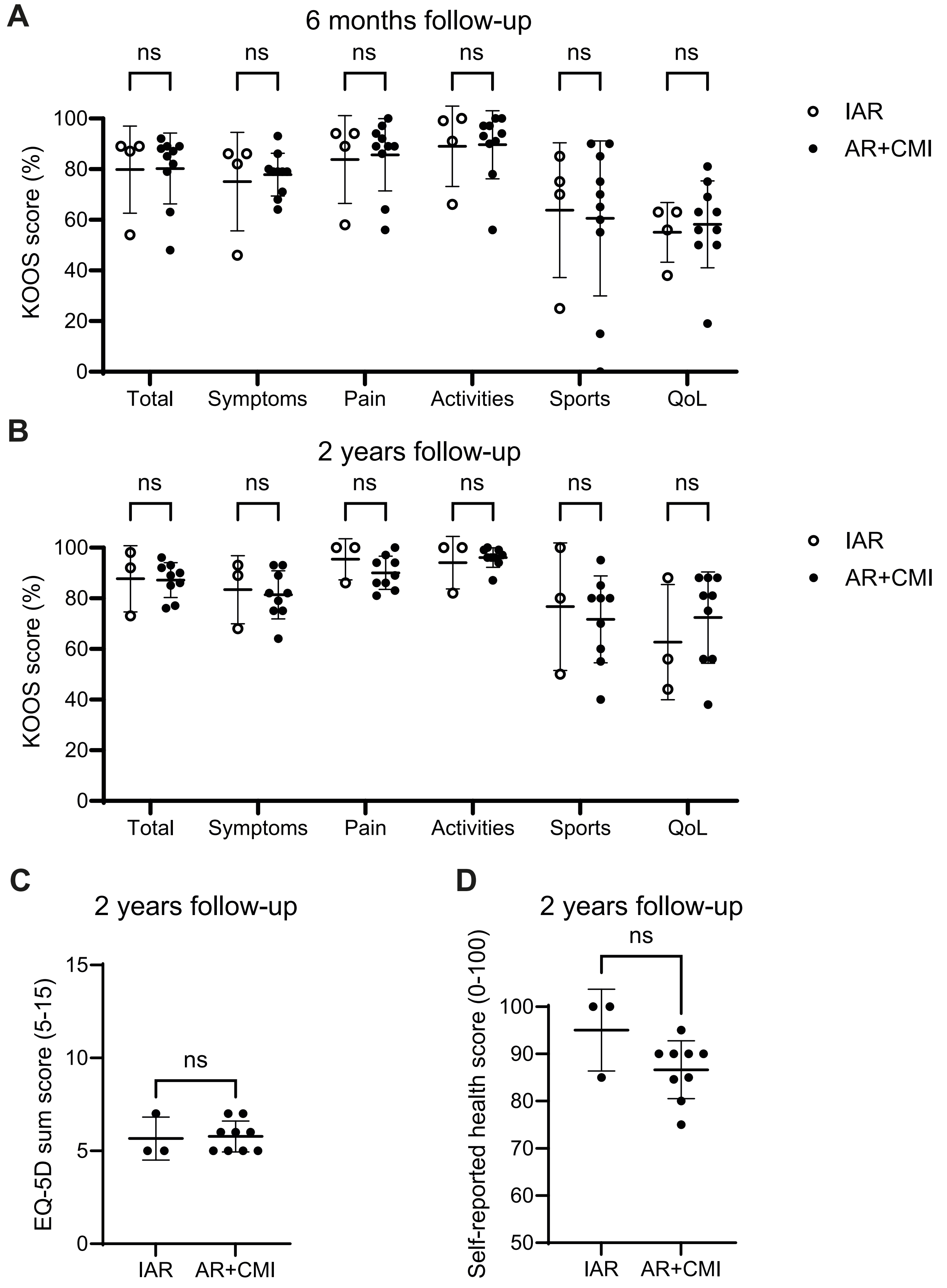

Additional file 9: Absolute follow-up KOOS and EQ-5D scores. A) Total KOOS score and scores of the individual KOOS subcategories 6 months and **B)** 2 years postoperatively. No differences were found between patients with an isolated ACL rupture and patients with concurrent meniscal injury. **C)** EQ-5D sum score and the **D)** self-reported health score 2 years postoperatively. No differences were found between the IAR and AR+CMI groups. KOOS 6 months follow-up: n=14 patients (IAR: n=4, AR+CMI: n=10); PROMs 2 years follow-up: n=12 patients (IAR: n=3, AR+CMI: n=9). Horizontal and vertical bars represent mean and standard deviation, respectively. KOOS = Knee Injury and Osteoarthritis Outcome Score; QoL = Quality of Life; IAR = isolated ACL rupture; AR+CMI = ACL rupture + concurrent meniscal injury. ns = not significant.
