# Supplementary material for: Cellular and molecular landscapes of inflammation in anterior cruciate ligament rupture patients are independent on concurrent meniscal injury
Source: Arthritis Res Ther. 2026 Apr 18;28:121. doi: 10.1186/s13075-026-03810-0 (PMC13220405; doi:10.1186/s13075-026-03810-0)
Supplement: Supplementary file 10 — Additional File 10: Correlations between histology and demographic data. A) Correlation matrix between histology scores, BMI and age. No correlations were found between the histology scores and BMI or age. B) No differences were found between males and females regarding histology scores. Horizontal and vertical bars represent mean and standard deviation, respectively. BMI = body mass index; ns = not significant [file 13075_2026_3810_MOESM10_ESM.pdf]

A

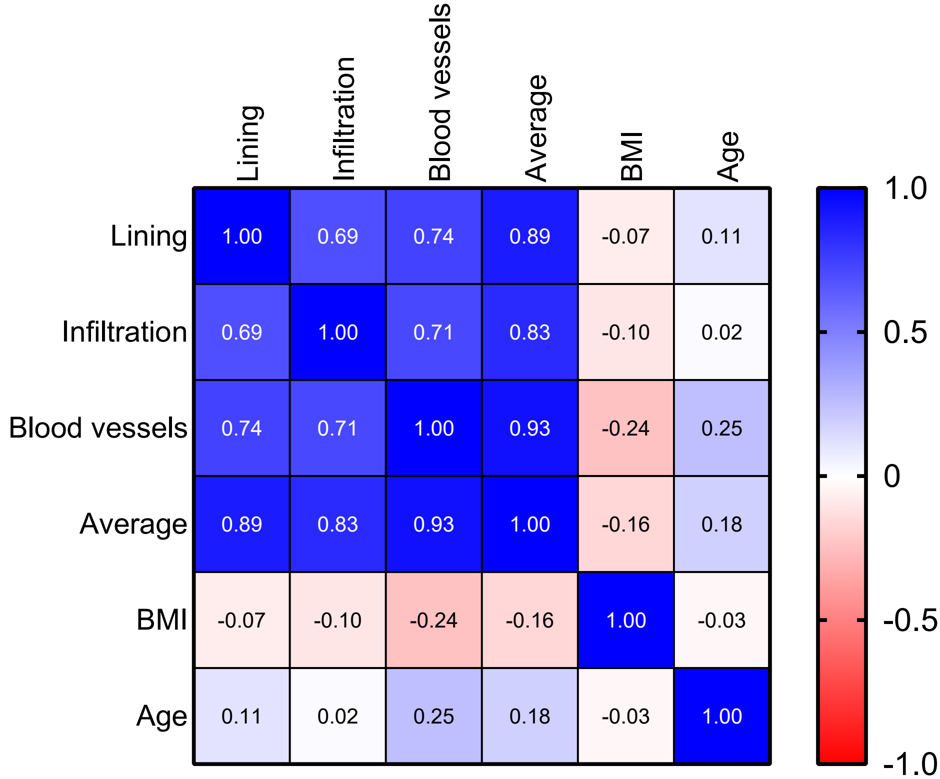

P values	**Lining**	**Infiltration**	**Blood vessels**	**Average**	**BMI**	**Age**
**Lining**		0.0002	0.00004	5.2114e-009	0.7404	0.6132
**Infiltration**	0.0002		0.00009	4.5932e-007	0.6451	0.9406
**Blood vessels**	3.8253e-005	0.00009		2.8531e-011	0.2574	0.2427
**Average**	5.2114e-009	4.5932e-007	2.8531e-011		0.4592	0.3917
**BMI**	0.7404	0.6451	0.2574	0.4592		0.8824
**Age**	0.6131	0.9406	0.2427	0.3917	0.8824	

B

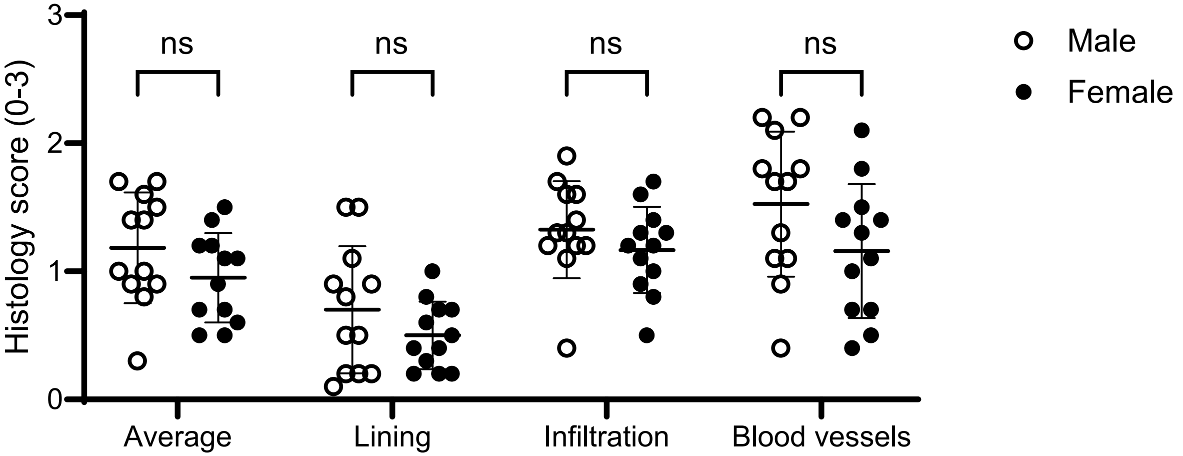

Additional file 10: Correlations between histology and demographic data. A) Correlation matrix between histology scores, BMI and age. No correlations were found between the histology scores and BMI or age. **B)** No differences were found between males and females regarding histology scores. Horizontal and vertical bars represent mean and standard deviation, respectively. BMI = body mass index; ns = not significant.
