# Supplementary material for: Cellular and molecular landscapes of inflammation in anterior cruciate ligament rupture patients are independent on concurrent meniscal injury
Source: Arthritis Res Ther. 2026 Apr 18;28:121. doi: 10.1186/s13075-026-03810-0 (PMC13220405; doi:10.1186/s13075-026-03810-0)
Supplement: Supplementary file 11 — Additional File 11: Correlations between BMI at baseline and Δ PROM scores at six months and two years follow-up in the AR+CMI group [file 13075_2026_3810_MOESM11_ESM.pdf]

Additional File 11: Correlations between BMI at baseline and Δ PROM scores at six months and two years follow-up in the AR+CMI group

Parameter correlated with BMI	r	P value
6M ΔTotal KOOS	0.01	1.00
6M ΔKOOS Symptoms	0.21	0.55
6M ΔKOOS Pain	0.01	1.00
6M ΔKOOS Activities	0.12	0.76
6M ΔKOOS Sports	-0.26	0.46
6M ΔKOOS QoL	-0.19	0.60
2Y ΔTotal KOOS	-0.13	0.74
2Y ΔKOOS Symptoms	0.08	0.85
2Y ΔKOOS Pain	-0.08	0.84
2Y ΔKOOS Activities	-0.05	0.92
2Y ΔKOOS Sports	-0.17	0.68
2Y ΔKOOS QoL	0.04	0.92
2Y ΔEQ-5D sum score	0.24	0.85
2Y ΔSelf-reported health score	0.07	0.54

BMI = Body Mass Index; PROM = Patient-reported Outcome Measures; AR+CMI = ACL rupture + concurrent meniscal injury; KOOS = Knee Injury and Osteoarthritis Outcome Score; QoL = Quality of Life
